# Pollination Mode and Mating System Explain Patterns in Genetic Differentiation in Neotropical Plants

**DOI:** 10.1371/journal.pone.0158660

**Published:** 2016-07-29

**Authors:** Liliana Ballesteros-Mejia, Natácia E. Lima, Matheus S. Lima-Ribeiro, Rosane G. Collevatti

**Affiliations:** 1 Laboratório de Genética & Biodiversidade, Instituto de Ciências Biológicas, Universidade Federal de Goiás, Cx.P. 131, 74001–970, Goiânia, GO, Brazil; 2 Laboratório de Macroecologia, Universidade Federal de Goiás, Campus Jataí, Cx.P. 03, 75801–615, Jataí, GO, Brazil; University of Northampton, UNITED KINGDOM

## Abstract

We studied genetic diversity and differentiation patterns in Neotropical plants to address effects of life history traits (LHT) and ecological attributes based on an exhaustive literature survey. We used generalized linear mixed models (GLMMs) to test the effects as fixed and random factors of growth form, pollination and dispersal modes, mating and breeding systems, geographical range and habitat on patterns of genetic diversity (*H*_*S*_, *He*_*S*_, *π* and *h*), inbreeding coefficient (*F*_*IS*_), allelic richness (*AR*) and differentiation among populations (*F*_*ST*_) for both nuclear and chloroplast genomes. In addition, we used phylogenetic generalized least squares (pGLS) to account for phylogenetic independence on predictor variables and verify the robustness of the results from significant GLMMs. In general, GLMM revealed more significant relationships among LHTs and genetic patterns than pGLS. After accounting for phylogenetic independence (i.e., using pGLS), *F*_*ST*_ for nuclear microsatellites was significantly related to pollination mode, mating system and habitat. Plants specifically with outcrossing mating system had lower *F*_*ST*_. Moreover, *AR* was significantly related to pollination mode and geographical range and *He*_*S*_ for nuclear dominant markers was significantly related to habitat. Our findings showed that different results might be retrieved when phylogenetic non-independence is taken into account and that LHTs and ecological attributes affect substantially the genetic pattern in Neotropical plants, hence may drive key evolutionary processes in plants.

## Introduction

The search for patterns in evolutionary ecology has been extensively discussed in literature as a central problem in ecology (see [1,2] for recent reviews). Lawton ([3] p 145) stated, “Without bold, regular patterns in nature, ecologists do not have anything very interesting to explain”. However, the observed variables and the ecological and evolutionary mechanisms affecting the patterns operate at different scales across space, time and ecological organization, challenging the finding of such patterns and their ecological and evolutionary causes [4]. In population genetics, genetic diversity and its distribution both within and among populations may be determined by microevolutionary processes such as demographic history, selection and gene flow which in turn may operate at different scales of space, time and ecological organization (see [5] for a review). Studying this feature should then be the first step to understand the evolutionary path that a species undertake. Plant life-history traits (hereafter LHT) such as growth form, pollen and seed dispersal modes and breeding system, as well as geographical distribution and other ecological attributes may also influence genetic structure, gene flow and effective population size. As a consequence, life-history may determine the relative importance of microevolutionary processes, thus affecting population genetic structure [6].

Previous reviews in population genetics aiming to find patterns in genetic diversity and population structure in plants have shown relationship between LHT and population differentiation for isozyme/allozyme loci and dominantly inherited nuclear DNA markers such as RAPD, ISSR and AFLP [6–12]. These studies indicate that either long-lived woody or outcrossing species have higher genetic diversity within than among populations, contrary to annual selfing species. Long-lived outcrossing species may have larger effective population sizes preventing the loss of genetic diversity and population differentiation due to genetic drift [6]. Genetic diversity is significantly higher in larger populations, mainly in self-incompatible species, but the level of inbreeding *F*_*IS*_ is independent of population size [13]. In addition, due to low population density, significant rates of self-fertilization and biparental inbreeding (outcross between related individuals), tropical trees have significantly higher genetic differentiation than temperate forest trees [14]. Patterns in plant genetic diversity are also related to habitat fragmentation, still LHT such as pollination and seed dispersal modes are not related to the susceptibility to the loss of genetic diversity [15].

**Fig 1:**
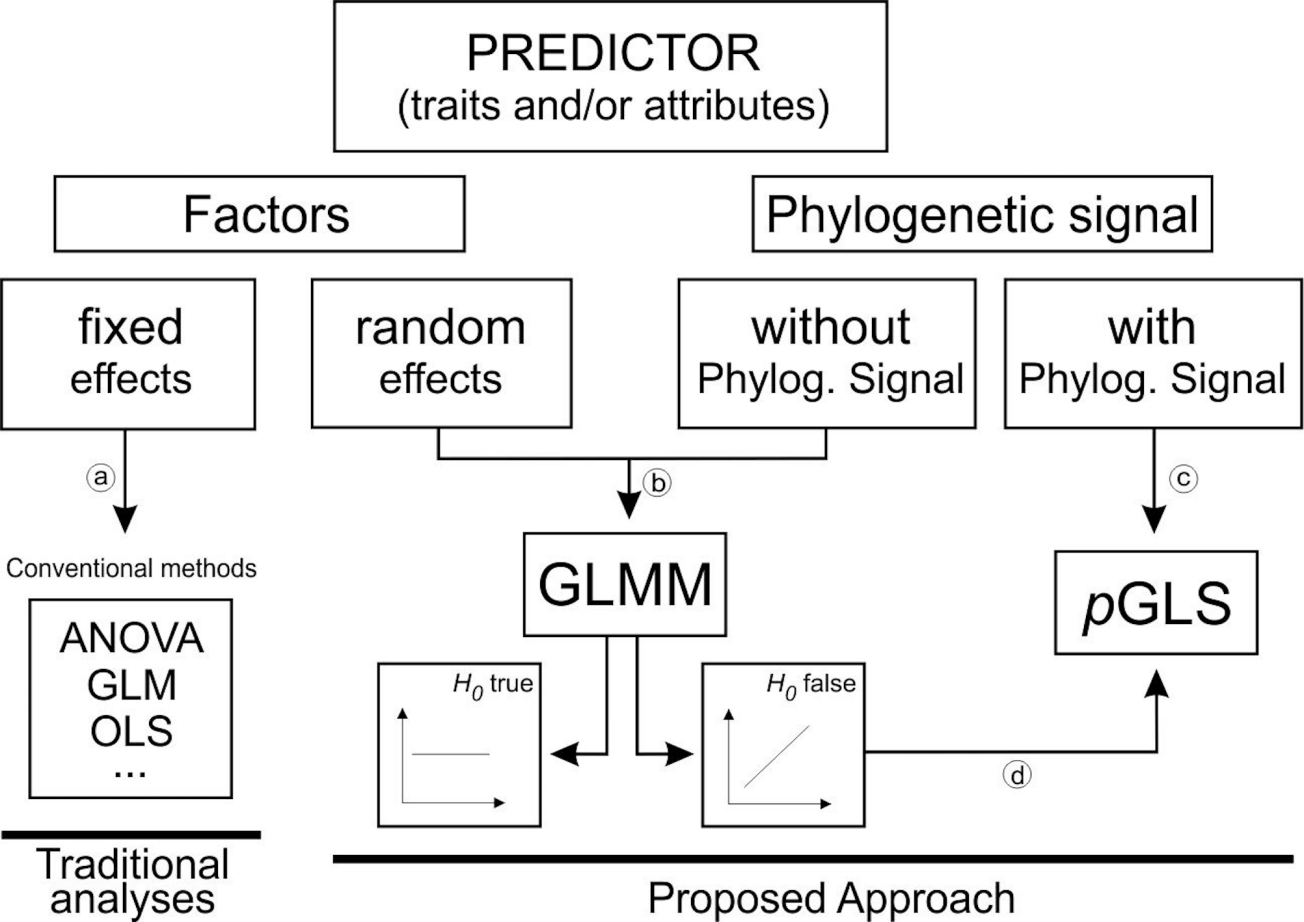
Conceptual framework summarizing the traditional analyses and the advanced approach proposed here to account for random factors and phylogenetic signal across predictors.

In general, the findings from these reviews reveal important patterns and their causes. However, they suffer serious statistical flaws. In this type of reviews, data is often non-normal distributed and researchers usually transform data to achieve normality and homogeneity of variance and rely on the robustness of ANOVA or use Generalized Linear Models (GLM) [8,9,16–18], (Fig 1, arrow “a”). These approaches may lead to errors since they ignore random effects from different LHT and treat all of them as fixed factors committing pseudoreplication (see [19,20] for a review). A more appropriate method to analyze such data would be generalized linear mixed models (GLMMs) because it combines desirable properties of two statistical frameworks; i.e. linear mixed models, incorporating random effects, and GLM, which handles non-normal data [21]. GLMM could thus match better the structure of the data (Fig 1, arrow “b”).

Besides the mixed structure of the data, traditional reviews have also ignored the phylogenetic relationship among the species (but see [16,17]). Conventional methods and GLMMs do not account for phylogenetic dependence on the predictors such as LHT. Related species tend to resemble each other more than species randomly chosen from a phylogenetic tree, as a consequence of stochastic character evolution (Brownian motion) along a phylogeny (phylogenetic signal [22]). Thus, the phenotypes of a set of species may not represent independent samples from populations or lineages therefore such statistical methods may not be appropriate for comparative analysis and hypotheses testing about trait correlation across species [23] due to risk of inflating type I error. Thus GLMMs should be applied only if mixed factors (predictors) do not show phylogenetic signal (Fig 1, arrow “b”).

Actually, it is necessary to first test for phylogenetic signal in the studied traits and if they are statistically significant then apply phylogenetically based methods for comparisons and hypotheses testing [24,25] (Fig 1, arrow “c”). When phylogenetic relationships are accounted for, related species tend to have similar levels of genetic diversity and differentiation for nuclear [17] and chloroplast molecular markers [16]. For example, in European Temperate and Boreal-Temperate angiosperms were found significant effects of reproduction system, pollination and dispersal modes, successional status and geographical distribution on population differentiation. [16]. However, when phylogenetic non-independence was considered using Phylogenetic Independent Contrast [23], only seed mass and geographical distribution remained significant [16]. Similarly, significant relationships were found for growth form, plant size, perenniality, seed dispersal mode, seed mass, pollination mode and mating system with genetic differentiation [17]. Yet, only perenniality, breeding and mating systems showed significant relationship with genetic differentiation at nuclear genome when phylogenetic non-independence was considered [17].

It is fundamental that phylogenetic relationships are previously considered in such reviews and analyses. However, reliable estimates of phylogenetic signal depend on enough sample size, which is not always available throughout reviews. Small samples might bias such estimates by overspread or clumping species throughout phylogenies and undesirably reveal weak or strong phylogenetic signals, respectively. To solve the problem of small sample size, the effect of phylogenetic signal could be directly inferred from GLMM analyses. Because phylogenetic non-independence inflate type I error (i.e., reject null hypotheses more times than expected by chance), only the significant relationships from GLMMs could suffer phylogenetic effects. Then, the robustness of significant GLMM relationships (and not non-significant ones) should be tested in the light of phylogenetic methods as pGLS (Fig 1, arrow “d”).

The Neotropics is one of the most diverse ecozones in the world; this vast biodiversity is a large repository of genetic information and has been defined as an active center of evolution [26]. Around 37% of seed plant species in the world occur in the Neotropics [27]. It includes eight of the 25 megadiverse hotspots, and some of the most threatened hotspots [28]. Hence, detection of genetic diversity and differentiation patterns is essential to address future research and conservation strategies in the Neotropics. For instance, geographical restricted species (i.e., presenting small effective population sizes) might experience rapid evolutionary changes due to rapid changes in allelic frequencies (genetic drift and founder effect [6,28]). In addition, previous works have showed that long-lived woody species may have higher genetic diversity and lower differentiation among populations due to larger effective populations sizes [6], however, in Neotropical rainforest, canopy trees usually occur at low density raising the question whether this expectation would hold in the Neotropics.

Furthermore, in Angiospermae, organelle DNA is usually inherited maternally but nuclear DNA is biparentally inherited. As a result, nuclear markers are transmitted via pollen and seeds whereas maternally inherited markers are transmitted via seeds only. Additionally, because of the haploid nature and mode of inheritance, the effective population size of the nuclear genome is four times the size of the chloroplast genome, leading to a stronger effect of genetic drift on population genetic structure based on chloroplast data [29]. Thus, the comparative analysis of nuclear and organelle genomes, with different modes of inheritance, and mutation and evolutionary rates, may provide different patterns of genetic diversity and differentiation and still clarify the relative importance of pollen and seed flow on population differentiation [30].

Because of their underrepresentation in most review studies and their importance for conservation, here, we focused on Neotropical plants addressing the effects of life-history traits, geographical range and habitat on patterns in genetic diversity and differentiation based on a literature survey. We used GLMMs to test the effects of growth form, pollination and dispersal mode, mating system (mixed, outcrossing or selfing), breeding system (monoecious, dioecious, hermaphrodite), geographical range and habitat on patterns of genetic diversity (*He*_*S*_, *He*, *π and h*), inbreeding (*F*_*IS*_), allelic richness (*AR*) and differentiation among populations (*F*_*ST*_), for both nuclear and chloroplast genomes in Neotropical plants. In addition, we used phylogenetic generalized least squares (pGLS) to account for phylogenetic relationships and verify the robustness of the results found by significant GLMMs.

Many Neotropical trees are widespread and have outcrossing mating system [6,9], in addition, long-lived woody species have typically larger effective population sizes [6], therefore we hypothesized that this growth form (i.e. trees) will have significant higher genetic diversity and allelic richness, and lower *F*_*ST*_ and *F*_*IS*_ than the other growth forms. Similarly, we expect that species with long distance dispersal modes might have lower *F*_*ST*_ and *F*_*IS*,_ as well as species with long distance pollination modes.

## Material and Methods

### Database survey

We surveyed publications of ‘population genetics of Neotropical plants’ from the Thomson Reuters Web of Science database (http://apps.webofknowledge.com), using Web of Science platform (ISI hereafter), and Scopus Search (http://www.elsevier.com/online-tools/scopus). The survey was performed considering the availability of publications in both databases from 1945 (first register in ISI) to December 2013. We used the most frequent keywords in the area: "population genetics", "phylogeography", "mating system", "reproductive system", “breeding system”, and "genetic structure", combined with (AND) "Neotropical trees" and "Neotropical plants". Additional keywords such as “pollen dispersal” and “gene flow” retrieved no additional articles. We excluded phylogenetic studies, reviews and development of molecular markers such as microsatellite primer development or SNPs discovery. We included only Angiospermae due to the low number of Gymnospermae species in the Neotropics (they occur mostly in Patagonia, across southern Argentina and Chile, as well as in highlands across Andes and Mesoamerica). We considered as Neotropics the region comprising the Neotropical Floristic Kingdom [31], which includes southern Florida, lowlands in Mexico, Central America, Caribe and South America, excluding southernmost South America (south Argentina and Chile, mainly Patagonia) and Andean highlands.

### Life-history traits and ecological attributes

From each article we compiled information related to the studied species, number of individuals and populations, species life-history traits (i.e. dispersal and pollination modes, mating system, breeding system and growth form) and ecological attributes (i.e. geographical range and habitat). Data on LHT and attributes were obtained from the original articles or from articles about pollination system, seed dispersal or botanical reviews. We avoided creating many categories (for example mammalian seed dispersal by scatter-hoarding or endozoocory) due to the low sample size per category.

We categorized species growth form as epiphytes, herbs, non-woody scandent vines, palms, shrubs, trees and woody long-lived vines. For seed dispersal mode we found species dispersed by autochory, bats (predominantly dispersed by bats), birds, water (hydrocory), terrestrial mammals, mixed (birds and mammals including bats) and wind. For pollination mode, we classified species pollinated by bats, beetles, butterflies, flies, hummingbirds, large bees, moths, small bees, wasps and wind (S1 Table). For breeding system we found monoecious, dioecious and hermaphrodite species. For mating system we found species with outcrossing and mixed systems. Due to the low number of studies for some LHT, we joined the categories: herbs and non-woody scandent vines (herbs hereafter); trees and woody long-lived vines (trees); autochory and wind dispersal (wind); butterfly and moth pollination (Lepidoptera); large and small bees and wasps (Hymenoptera).

For ecological attributes, species geographical range was classified in two categories, narrow (species endemic to a specific habitat or species with restrict geographical range that occur in twenty or fewer localities) and widespread (S2 Table). Because this information is ambiguous in many studies we also checked geographical range using GBIF database (www.gbif.org). For habitat, we found species from deserts, grasslands, mangroves, mixed forests (i.e. species occurring in both rainforests and seasonally dry forests), savannas, seasonally dry forests (SDTFs hereafter), rainforests, rocky fields, rocky savannas and wetlands (S2 Table).

### Genetic data

To compile genetic data we first classified the studies according to the molecular marker (S3 Table): dominant nuclear markers (AFLP, ISSR, RAPD); nuclear microsatellites; isozyme/allozyme (isozyme hereafter); nuclear sequences (mainly nrDNA ITS); chloroplast sequences (intergenic spacers); chloroplast microsatellites. For chloroplast, those studies using CAPs (cleaved amplified polymorphism) and similar markers (such as chloroplast RFLP and AFLP) were grouped (CAPs hereafter) due to the similar genetic information provided.

For chloroplast microsatellites and CAPs we compiled only genetic differentiation (*F*_*ST*_). For nuclear dominant markers (AFLP, ISSR, RAPD); nuclear microsatellites and isozymes we obtained *F*_*ST*_, genetic diversity within population (i.e. *He* for articles that studied only one population), overall population genetic diversity (i.e. *He*_*S*_ for articles that included more than one population), defined by [32] as genetic diversity at species level. For nuclear microsatellites and isozymes we also obtained inbreeding coefficient (*F*_*IS*_) and allelic richness based on rarefaction (*AR*). For chloroplast and nuclear sequences we obtained *F*_*ST*_ and nucleotide (*π*) and haplotype (*h*) diversities. However *F*_*ST*_ for nuclear sequences could not be analyzed due to the low sample size. Number of alleles and haplotypes were not analyzed due to the effect of sample size. Since articles not always reported the same genetic parameters, data set could vary among parameters (see Tables 1, 2 and 3).

**Table 1:** Mean values of genetic diversity and differentiation in Neotropical plants per life-history trait (LHT) and ecological attribute, across all the studies included in the analyses, for nuclear molecular markers. *N*, number of species analyzed; *F*_*ST*_, genetic differentiation among populations; *He*_*S*_, overall population genetic diversity, *He*, genetic diversity within population; SD, standard deviation; n, sample size (number of species with available data in the regression analysis).

Life-history trait and ecological attribute		N	Dominant *F*_*ST*_ ± SD(n)	Microsatellites *F*_*ST*_ ± SD(n)	Isozymes *F*_*ST*_ ± SD(n)	Dominant *He*_*S*_ ± SD(n)	Isozymes *He*_*S*_ ± SD(n)	Microsatellites *He* ± SD(n)	Isozymes *He* ± SD(n)
**Growth form**	**Epiphytes**	17	0.177±0.023(4)	0.307±0.239(7)	0.45±0.310(2)	0.157±0.024(4)	0.183±0.005(2)	-	-
	**Herbs**	25	0.141±0.117(6)	0.015±0.000(1)	0.28±0.000(1)	0.520±0.255(2)	0.056±0.000(1)	-	-
	**Palms**	14	0.147±0.143(7)	0.170±0.248(4)	0.08±0.070(4)	0.552±0.286(6)	0.385±0.044(4)	-	0.371±0.000(1)
	**Shrubs**	21	0.383±0.226(5)	-	0.07±0.030(4)	0.300±0.053(3)	0.312±0.128(4)	-	0.141±0.000(1)
	**Trees**	109	0.202±0.152(35)	0.220±0.189(29)	0.15±0.110(20)	0.248±0.052(2)	0.413±0.172(12)	0.752±0.131(7)	0.325±0.168(2)
**Dispersal mode**	**Bats**	2	-	-		-	-	-	0.205±0.000(1)
	**Birds**	34	0.217±0.219(9)	0.311±0.291(7)	0.14±0.190(10)	0.209±0.109(5)	0.361±0.097(11)	-	0.256±0.163(2)
	**Hydrochory**	7	0.073±0.064(2)	0.453±0.057(5)	-	0.248±0.052(2)	-	-	-
	**Mammals**	60	0.198±0.138(25)	0.171±0.152(14)	0.19±0.130(11)	0.530±0.258(7)	0.515±0.210(4)	0.683±0.134(4)	0.444±0.000(1)
	**Mixed**	3	0.062±0.000(1)	-	0.12±0.000(1)	-	0.443±0.000(1)	-	-
	**Wind**	72	0.222±0.160(20)	0.159±0.168(15)	0.13±0.090(9)	0.374±0.285(3)	0.241±0.160(7)	0.845±0.039(3)	-
**Pollination mode**	**Bats**	18	0.090±0.000(1)	0.060±0.047(4)	0.17±0.070(2)	-	0.806±0.000(1)	0.627±0.119(2)	-
	**Beetles**	2	-	0.239±0.000(1)	0.13±0.000(1)	-	0.357±0.000(1)	-	-
	**Flies**	2	0.158±0.001(2)	-		0.137±0.001(2)	-	-	-
	**Hymenoptera**	130	0.224±0.163(47)	0.235±0.201(28)	0.13±0.110(24)	0.419±0.259(11)	0.360±0.117(17)	0.803±0.105(5)	0.290±0.141(4)
	**Hummingbirds**	18	0.082±0.101(4)	0.068±0.075(2)	0.45±0.310(2)	0.254±0.060(2)	0.140±0.073(3)	-	-
	**Lepidoptera**	14	0.139±0.030(2)	0.340±0.244(6)	0.06±0.000(1)	0.284±0.000(1)	0.448±0.000(1)	-	-
	**Wind**	4	0.020±0.000(1)	-	0.28±0.000(1)	0.700±0.000(1)	-	-	-
**Mating system**	**Mixed**	62	0.243±0.123(19)	0.339±0.216(16)	0.33±0.150(8)	0.281±0.179(6)	0.294±0.182(6)	0.733±0.167(3)	-
	**Outcrossing**	124	0.195±0.179(33)	0.152±0.157(25)	0.09±0.070(21)	0.448±0.271(10)	0.367±0.162(15)	0.767±0.122(4)	0.290±0.141(4)
**Breeding system**	**Dioecious**	19	0.132±0.111(5)	0.141±0.090(2)	0.09±0.060(5)	-	0.405±0.048(5)	0.622±0.000(1)	0.205±0.000(1)
	**Monoecious**	25	0.190±0.175(11)	0.195±0.180(8)	0.03±0.020(2)	0.552±0.286(6)	0.389±0.000(1)	-	0.371±0.000(1)
	**Hermaphrodite**	142	0.215±0.159(41)	0.239±0.214(31)	0.18±0.150(23)	0.278±0.159(11)	0.334±0.191(16)	0.774±0.128(6)	0.292±0.214(2)
**Habitat**	**Deserts**	3	-	0.015±0.000(1)		-	-	-	-
	**Grasslands**	8	0.060±0.000(3)	-		0.770±0.000(3)	-	-	-
	**Mangroves**	6	0.027±0.000(1)	0.453±0.057(5)		0.211±0.000(1)	-	-	-
	**Mixed**	10	0.358±0.109(7)	0.042±0.000(1)	0.23±0.12(6)	0.119±0.000(1)	0.395±0.008(2)	0.856±0.000(1)	0.371±0.000(1)
	**Rainforests**	71	0.120±0.140(15)	0.163±0.136(18)	0.17±0.190(11)	0.203±0.072(5)	0.354±0.135(10)	0.739±0.165(2)	0.325±0.169(2)
	**Rocky fields**	8	0.559±0.000(1)	0.650±0.000(2)	0.06±0.050(2)	0.250±0.000(1)	0.223±0.169(3)	-	-
	**Rocky savannas**	4	0.344±0.000(1)	0.712±0.000(1)		-	-	-	-
	**Savannas**	16	0.194±0.054(10)	0.190±0.198(6)	0.17±0.09(3)	0.296±0.000(1)	0.581±0.317(2)	0.543±0.000(1)	-
	**Seasonally dry forests**	57	0.228±0.181(16)	0.118±0.051(7)	0.10±0.080(9)	0.500±0.180(4)	0.332±0.139(6)	0.797±0.084(3)	0.141±0.000(1)
	**Wetlands**	3	0.184±0.056(3)	-		0.171±0.000(1)	-	-	-
**Geographical range**	**Narrow**	71	0.177±0.177(20)	0.277±0.277(12)	0.23±0.18(12)	0.535±0.273(7)	0.272±0.149(7)	-	-
	**Widespread**	115	0.217±0.147(37)	0.204±0.163(29)	0.11±0.090(19)	0.263±0.149(10)	0.391±0.159(16)	0.752±0.131(7)	0.290±0.141(4)

**Table 2:** Mean values of genetic diversity and differentiation in Neotropical plants per life-history trait (LHT) and ecological attribute, across all the studies included in the analyses, for nuclear molecular markers. *N*, number of species analyzed; *F*_*IS*_, inbreeding coefficient; *AR*, allelic richness; *h*, haplotype diversity; π, nucleotide diversity; SD, standard deviation; n, sample size (number of species with available data in the regression analysis).

Life-history trait and ecological attribute		N	Isozymes *F*_*IS*_ ±	Microsatellites	Isozymes *AR* ±	Microsatellites	Nuclear sequences	
			SD(n)	*F*_*IS*_ ± SD(n)	SD(n)	*AR* ± SD(n)	*h* ± SD(n)	*π* ± SD(n)
**Growth form**	**Epiphytes**	17	0.368±0.387(2)	0.101±0.091(10)	1.330±0.113(2)	5.243±2.695(6)	-	-
	**Herbs**	25	0.127±0.000(1)	0.057±0.076(2)	-	3.060±0.000(1)	0.841±0.000(1)	0.005±0.000(3)
	**Palms**	14	0.099±0.363(5)	0.229±0.152(3)	1.705±0.177(2)	2.350±0.000(1)	-	-
	**Shrubs**	21	0.023±0.426(4)	0.125±0.000(1)	-	2.320±0.000(1)	0.764±0.050(2)	0.008±0.007(2)
	**Trees**	109	0.021±0.145(21)	0.099±0.090(33)	1.659±0.541(7)	5.679±3.514(23)	0.894±0.050(6)	0.012±0.011(11)
**Dispersal mode**	**Bats**	2	0.240±0.000(1)	-	-	-	-	-
	**Birds**	34	0.054±0.363(11)	0.148±0.126(7)	1.767±0.481(5)	4.973±3.021(5)	0.801±0.000(1)	0.008±0.006(2)
	**Hydrochory**	7	-	0.115±0.148(2)	-	1.680±0.000(2)	0.867±0.000(3)	0.013±0.000(3)
	**Mammals**	60	0.018±0.137(12)	0.097±0.083(18)	1.587±0.603(3)	6.240±4.242(11)	0.958±0.000(2)	0.010±0.010(5)
	**Mixed**	3	0.133±0.000(1)	-	-	-	0.841±0.000(1)	0.006±0.000(1)
	**Wind**	72	0.089±0.204(8)	0.100±0.095(22)	1.363±0.117(3)	5.210±2.493(14)	0.787±0.090(2)	0.010±0.015(5)
**Pollination mode**	**Bats**	18	0.106±0.000(1)	0.094±0.086(10)	-	3.940±1.157(3)	-	0.004±0.003(2)
	**Beetles**	2	0.017±0.000(1)	0.121±0.00(1)	-	14.330±0.000(1)	-	-
	**Flies**	8	-	-	-	-	-	-
	**Hymenoptera**	130	0.066±0.210(25)	0.117±0.106(29)	1.669±0.473(9)	4.857±2.890(22)	0.880±0.058(7)	0.012±0.010(12)
	**Hummingbirds**	18	0.288±0.307(3)	0.064±0.087(2)	1.330±0.113(2)	10.590±0.000(1)	-	-
	**Lepidoptera**	14	-0.251±0.305(3)	0.087±0.078(7)	-	5.241±3.013(5)	0.787±0.086(2)	0.002±0.001(2)
	**Wind**	4	-	-	-	-	-	-
**Mating system**	**Mixed**	62	0.163±0.249(7)	0.086±0.097(15)	1.757±0.608(5)	4.612±3.043(12)	0.874±0.085(6)	0.014±0.006(6)
	**Outcrossing**	124	0.038±0.243(24)	0.115±0.095(34)	1.483±0.248(6)	5.723±3.423(20)	0.821±0.028(2)	0.008±0.011(9)
**Breeding system**	**Dioecious**	19	0.141±0.328(6)	0.154±0.217(3)	1.705±0.177(2)	4.178±2.146(2)	-	0.003±0.002(2)
	**Monoecious**	25	0.013±0.126(3)	0.129±0.089(4)	1.240±0.085(2)	4.468±3.644(4)	0.958±0.000(2)	0.020±0.000(2)
	**Hermaphrodite**	142	0.042±0.239(23)	0.100±0.087(41)	1.685±0.520(7)	5.522±3.363(26)	0.831±0.052(7)	0.009±0.010(12)
**Habitat**	**Deserts**	3	0.057±0.080(3)	0.057±0.076(2)	-	3.060±0.000(1)	-	-
	**Grasslands**	8	-	-	-	-	-	-
	**Mangroves**	6	0.115±0.150(3)	0.115±0.148(2)	-	1.680±0.000(2)	0.867±0.000(3)	0.013±0.000(3)
	**Mixed**	10	0.054±0.130(3)	0.162±0.117(2)	-	8.340±2.220(2)	-	-
	**Rainforests**	71	0.088±0.210(17)	0.098±0.089(22)	1.579±0.388(7)	5.814±2.891(13)	0.958±0.000(1)	0.009±0.010(4)
	**Rocky fields**	8	0.218±0.120(3)	0.175±0.093(2)	-	3.455±0.361(2)	-	-
	**Rocky savannas**	4	-	-0.021±0.00(1)	-	1.800±0.00(1)	0.726±0.000(1)	0.003±0.00(1)
	**Savannas**	16	0.106±0.090(2)	0.100±0.091(8)	-	6.578±5.482(5)	-	0.001±0.000(1)
	**Seasonally dry forests**	57	0.053±0.160(8)	0.126±0.117(10)	1.658±0.598(4)	4.922±2.326(6)	0.862±0.067(4)	0.012±0.010(7)
	**Wetlands**	3	-	-	-	-	-	-
**Geographical range**	**Narrow**	71	0.123±0.293(9)	0.083±0.095(15)	1.330±0.113(2)	3.761±1.881(10)	0.787±0.090(2)	0.003±0.007(2)
	**Widespread**	115	0.033±0.223(24)	0.117±0.096(34)	1.669±0.473(9)	6.009±3.567(22)	0.880±0.060(7)	0.011±0.010(14)

**Table 3:** Mean values of genetic diversity and differentiation in Neotropical plants per life-history trait (LHT) and ecological attribute across all the studies included in the analyses, for chloroplast molecular markers. *N*, number of species analyzed; *F*_*ST*_, genetic differentiation among populations; *h*, haplotype diversity; π, nucleotide diversity; SD, standard deviation; n, sample size (number of species with available data in the regression analysis).

Life-history trait and ecological attribute		N	*F*_*ST*_ ± SD(n)	*h* ± SD(n)	*π* ± SD(n)
**Growth form**	**Epiphytes**	17	0.567±0.166(6)	0.589±0.066(2)	-
	**Herbs**	25	0.710±0.326(7)	0.618±0.277(9)	0.001±0.0005(7)
	**Palms**	14	0.880±0.000(1)	0.786±0.000(1)	0.004±0.0000(1)
	**Shrubs**	21	0.884±0.046(7)	0.603±0.248(4)	0.002±0.0020(3)
	**Trees**	109	0.628±0.305(32)	0.452±0.333(5)	0.007±0.0150(24)
**Dispersal mode**	**Bats**	2	-	-	-
	**Birds**	34	0.585±0.182(4)	0.535±0.116(2)	0.0019±0.0020(3)
	**Hydrochory**	7	-	0.695±0.000(3)	0.005±0.0001(3)
	**Mammals**	60	0.651±0.332(12)	0.867±0.114(2)	0.011±0.0220(10)
	**Mixed**	3	-	-	-
	**Wind**	72	0.686±0.279(37)	0.522±0.292(14)	0.003±0.0040(19)
**Pollination mode**	**Bats**	18	0.530±0.389(6)	-	0.017±0.0320(5)
	**Beetles**	2	-	-	-
	**Flies**	8	-	-	-
	**Hymenoptera**	130	0.805±0.227(31)	0.593±0.171(15)	0.001±0.0004(25)
	**Hummingbirds**	18	0.670±0.297(8)	0.585±0.303(3)	0.004±0.0036(2)
	**Lepidoptera**	14	0.642±0.138(8)	0.546±0.132(3)	0.005±0.0040(3)
	**Wind**	4	-	-	-
**Mating system**	**Mixed**	62	0.663±0.256(17)	0.656±0.166(7)	0.004±0.0010(7)
	**Outcrossing**	124	0.669±0.297(31)	0.578±0.281(13)	0.006±0.0140(26)
**Breeding system**	**Dioecious**	19	0.610±0.382(2)	0.786±0.000(1)	0.006±0.0030(3)
	**Monoecious**	25	0.680±0.286(8)	0.551±0.258(1)	0.0055±0.0130(1)
	**Hermaphrodite**	142	0.634±0.284(43)	0.937±0.000(19)	0.004±0.0001(31)
**Habitat**	**Deserts**	3	0.907±0.000(1)	0.095±0.000(1)	-
	**Grasslands**	8	0.853±0.122(3)	0.673±0.164(5)	0.001±0.0005(5)
	**Mangroves**	6	-	0.695±0.000(3)	0.005±0.0001(3)
	**Mixed**	10	-	-	-
	**Rainforests**	71	0.627±0.288(15)	0.528±0.356(4)	0.016±0.0330(5)
	**Rocky fields**	8	0.663±0.092(3)	0.583±0.338(2)	0.001±0.0006(2)
	**Rocky savannas**	4	0.399±0.403(2)	0.395±0.000(1)	0.004±0.0010(2)
	**Savannas**	16	0.823±0.147(3)	0.947±0.000(1)	0.006±0.0030(3)
	**Seasonally dry forests**	57	0.661±0.310(25)	0.416±0.286(3)	0.005±0.0040(14)
	**Wetlands**	3	0.880±0.000(1)	0.786±0.000(1)	0.004±0.0000(1)
**Geographical range**	**Narrow**	71	0.673±0.283(21)	0.564±0.231(11)	0.002±0.00180(15)
	**Widespread**	115	0.668±0.288(32)	0.600±0.306(10)	0.008±0.0160(20)

### Data Analysis

We fitted Generalized Linear Mixed Models (GLMMs) to investigate the effects of species LHT and ecological attributes on genetic diversity and differentiation. LHT (*i*.*e*. growth form, dispersal and pollination modes and breeding systems), and habitat type were treated as multi-state categorical variables, whereas geographical range (*i*.*e*. narrow or widespread) and mating system (*i*.*e*. mixed or outcrossing) were treated as binary variable. Models were fitted for each genetic parameter. Species LHT and ecological attributes were fitted as *fixed factors*, and species identity was considered a *random factor* as multiple variables were measured per species. Analyses were performed using *MCMCglmm* package [33] implemented in R version 3.2.1 (R core team 2014). *MCMCglmm* uses a Bayesian framework with Markov Chain Monte Carlo algorithm in which a total of 80,000 iterations chains were used with 2,000 chains of burn-in with a Gaussian distribution.

The effect of molecular markers on genetic parameters, was analyzed by fitting separate models for (1) nuclear dominant markers (i.e. AFLP, ISSR and RAPD); (2) isozymes; (3) nuclear microsatellites; (4) chloroplast markers (i.e. CAPs, chloroplast microsatellite and sequences). Due to the inheritance mode, chloroplast genome (uniparental) has 1/4 the effective population size of nuclear genome (biparental), which may affect the estimation of genetic variability and differentiation parameters, introducing noises in analyses precluding the detection of any pattern due to life-history traits or ecological attributes. When a species was studied more than once with the same molecular marker, the mean of the genetic parameters was used (40 cases in 186 species).

### Accounting for phylogenetic relationships

To account for phylogenetic non-independence on the effects of LHT and ecological attributes on genetic diversity and differentiation, we first obtained the reference phylogenetic hypothesis of the species included in each analysis. We used the internal master tree *Phylomatic tree R20120829* from the platform *Phylomatic* [34] to built the phylogenetic hypothesis. Since we had no information on branch length for inclusion in the comparative analyses, all branch lengths were assigned a value of 1, which may not significantly bias the results [35]. Even the exact phylogeny of some taxa is still in debate, improvements on the phylogeny would not modify dramatically the results because polytomies are mainly at terminal nodes and most of phylogenetic relationships are well resolved at deeper levels [36].

To account for phylogenetic relationships we first tested whether the studied life-history traits and ecological attributes have phylogenetic signal (i.e. phylogenetically related species tend to be more similar than distantly related species [22]). We used Abouheif’s proximity test of serial independence [37,38] using the function *abouheif*.*moran* from the R-package *adephylo* [39]

Then, we fitted Phylogenetic Generalized Least Square Models (pGLS [40]) to the genetic parameters verifying whether GLMM had resulted in robust inferences and hence the pattern persisted when phylogenetic relationships were accounted for. pGLS is a comparative method in which the covariance among species resulting from the phylogeny is expressed in the regression error term. It is easily extended to multivariate data and can be used unambiguously when polytomies are found in the phylogeny as in our case. The analyses were carried out using the package *caper* [41] implemented in R version 3.2.1.

## Results

### Data description

Our survey retrieved 358 articles comprising studies from 186 species, belonging to 45 families and 116 genera (S4 Table). Tree species were the most studied in Neotropics (109 species, S2 Table), followed by herbs (25 species), shrubs (21), epiphytes (17) and palms (14). Wind- and mammal-dispersed species were the most studied (81 and 60 species, respectively) and only two studied species were bat-dispersed. Most studied species were pollinated by Hymenoptera (i.e. large and small bees and wasps, 130 species), followed by Lepidoptera (butterflies and moths, 14) and wind-, fly- and beetle-pollinated species were underrepresented in the studies performed so far in Neotropical plants (S2 Table). For breeding system, hermaphrodite plants were the most studied (142), followed by monoecious (25) and dioecious (19). For mating system, plants with an outcrossing system (124) were the most studied (S2 Table).

Regarding the habitat and geographical range, most species studied up to now are widespread (115) across rainforests (71) and seasonally dry forests (57, S3 Table). The most used molecular marker (S4 Table) was nuclear microsatellites (61 species) followed by chloroplast sequences (51) and isozymes/allozymes (51).

We found high mean values and variation of *F*_*ST*_ for all LHT and ecological attributes for both nuclear and chloroplast molecular markers (Table 1). However, plants from deserts and grasslands had low values of *F*_*ST*_ at nuclear genome and plants pollinated by flies had high *F*_*ST*_ at chloroplast genome. The surveyed data also showed high variation in genetic diversity for both nuclear and chloroplast genomes (Tables 1, 2 and 3).

### Genetic patterns

#### General genetic patterns

**Fig 2:**
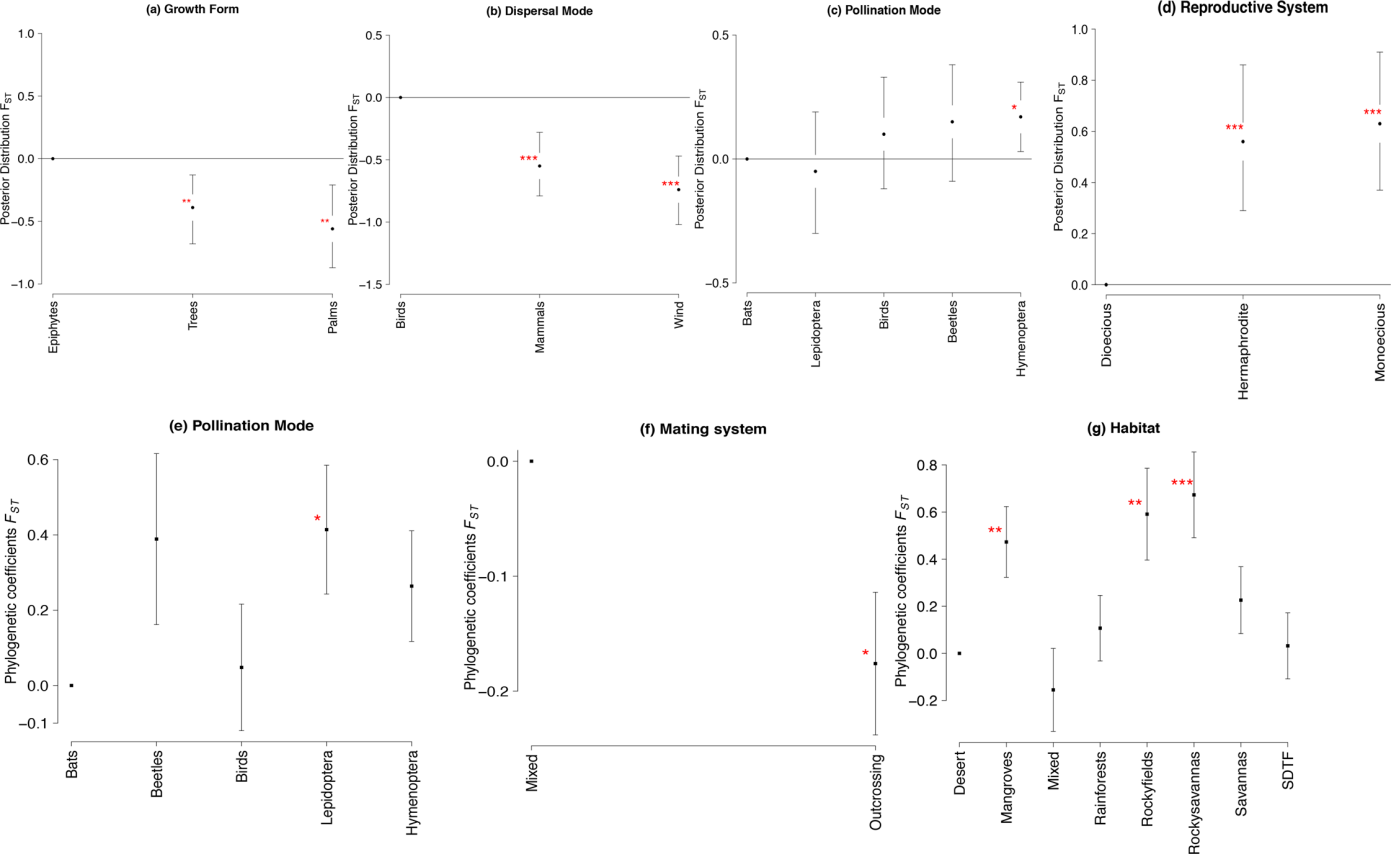
Mean values and 95% confidence intervals in the posterior distribution of the GLMM and phylogenetic coefficients and standard errors of pGLS for *F*_*ST*_ for nuclear microsatellite markers. (**a**) Growth form. (**b**) Dispersal mode. (**c**) Pollination mode. (**d)** Breeding system. **(e**) Breeding system phylogenetic coefficients. (**f**) Mating system phylogenetic coefficients. (**g**) Habitat phylogenetic coefficients. Values highlighted by an asterisk are significant (* 0.05<P<0.01, ** P< 0.01, ***P<0.000).

Genetic differentiation among populations (*F*_*ST*_) for nuclear microsatellite markers was significantly related to growth form, dispersal and pollination modes and breeding system (Fig 2, see also S5 Table). *F*_*ST*_ was significantly lower for trees and palms than for epiphytes, as well as lower for wind- and mammal-dispersed plants than for bird-dispersed (Fig 2A and 2B). In addition, *F*_*ST*_ values were significantly higher for plants pollinated by Hymenoptera and with monoecious and hermaphrodite breeding systems than for dioecious (Fig 2C and 2D, S5 Table). For chloroplast genome shrubs had higher *F*_*ST*_ than the other growth forms (S1 Fig, S6 Table).

**Fig 3:**
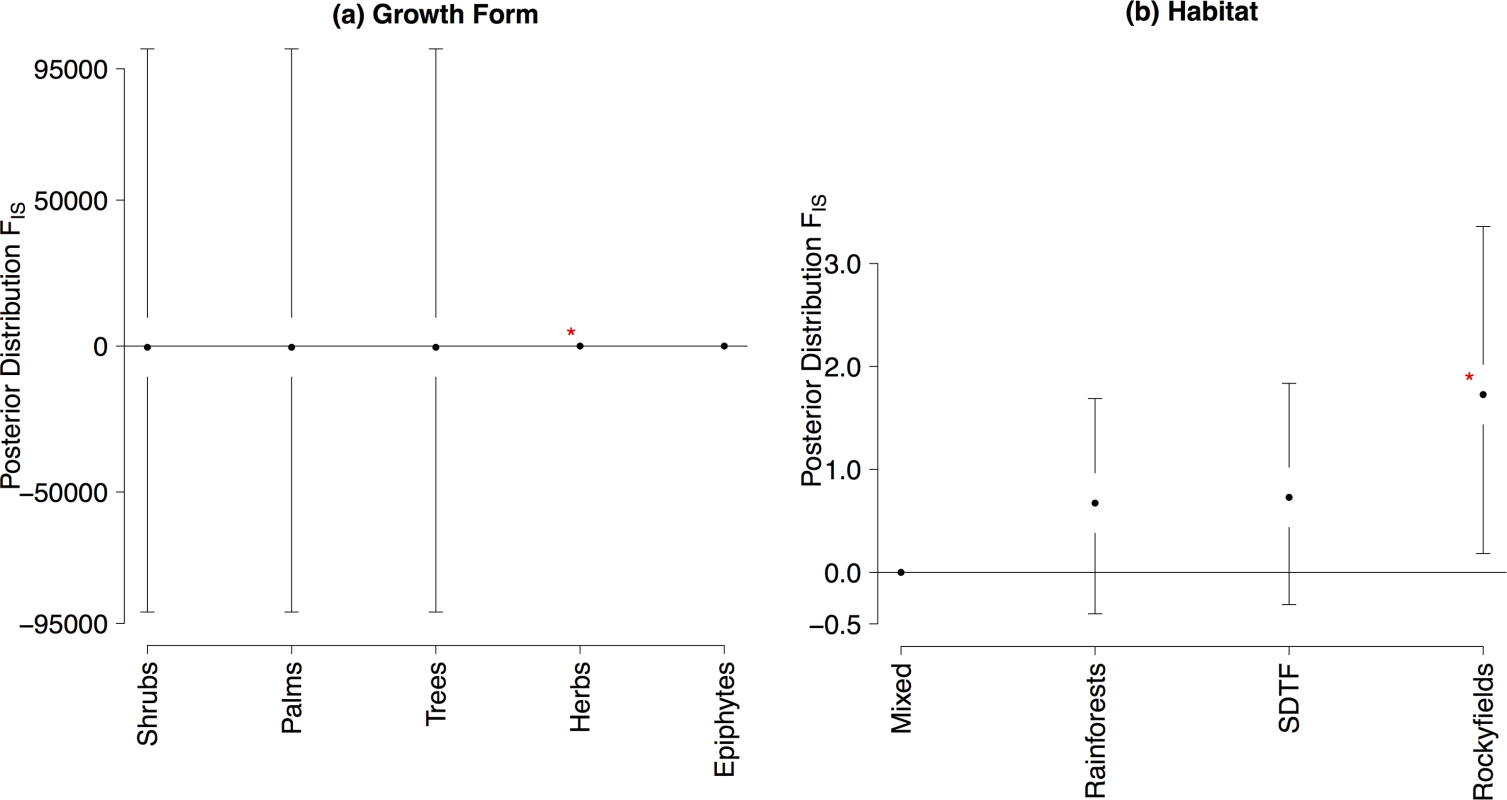
Mean values and 95% confidence intervals in the posterior distribution of the GLMM for *F*_*IS*_ retrieved from isozymes. (**a**) Growth form. (**b**) Habitat. Values highlighted by an asterisk are significant (* 0.05<P<0.01). Note that the mean value in posterior distribution for herbs in (a) -1.16.

Inbreeding coefficients (*F*_*IS*_) retrieved from isozymes were significantly higher for herbs than for the other growth forms (Fig 3A, S7 Table) and for plants inhabiting rocky fields than for plants from rainforests, mixed and SDTFs (Fig 3B, S7 Table).

**Fig 4:**
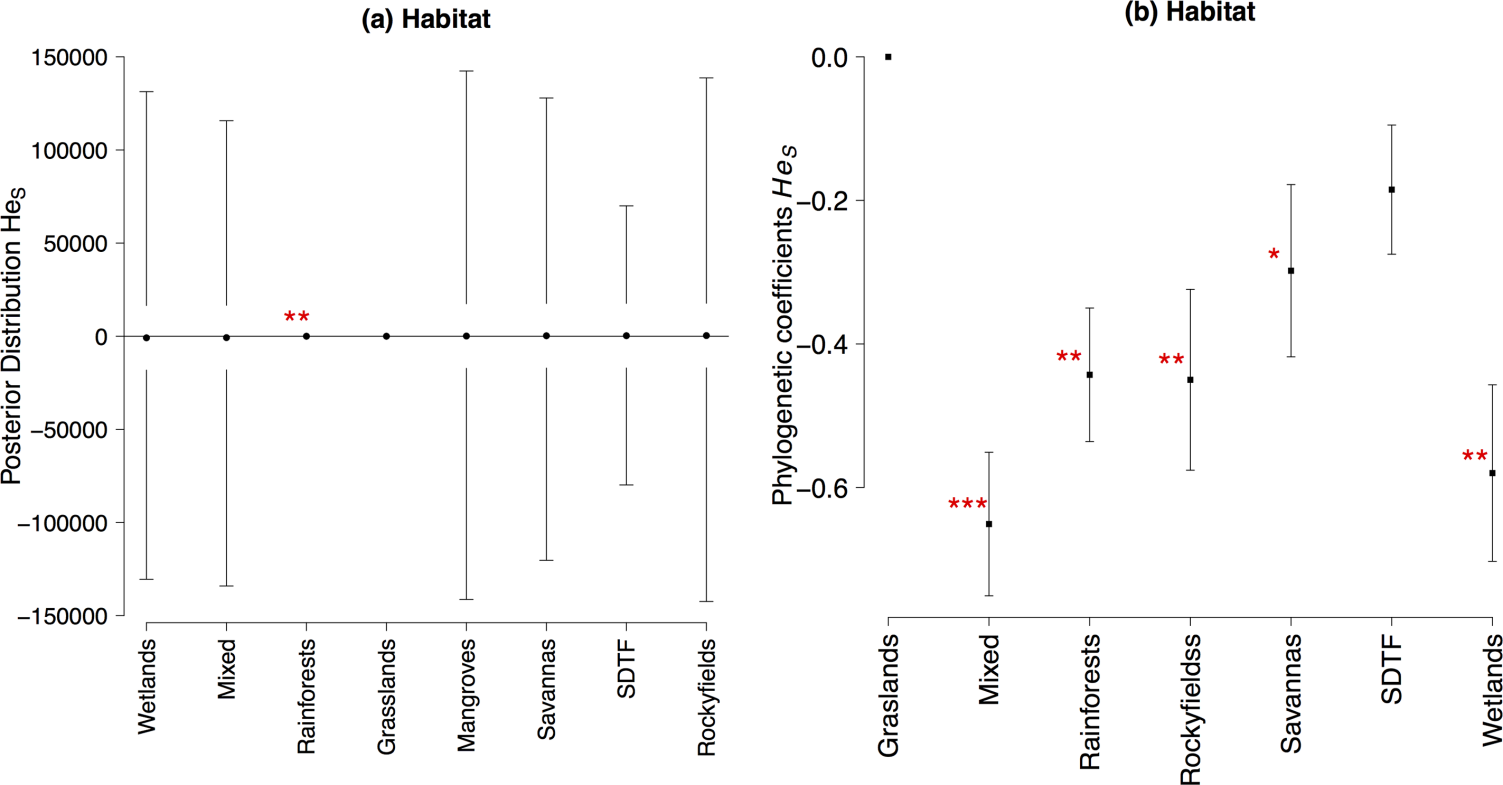
Mean values and 95% confidence intervals in the posterior distribution of the GLMM and phylogenetic coefficients and standard errors of pGLS for genetic diversity overall populations (*He*_*S*_) for nuclear dominant markers. (**a**) Habitat (**b**) Habitat phylogenetic coefficients. Values highlighted by an asterisk are significant (* 0.05<P<0.01, ** P< 0.01). Note that the mean value in posterior distribution for rainforests in (**a**) is -0.50.

**Fig 5:**
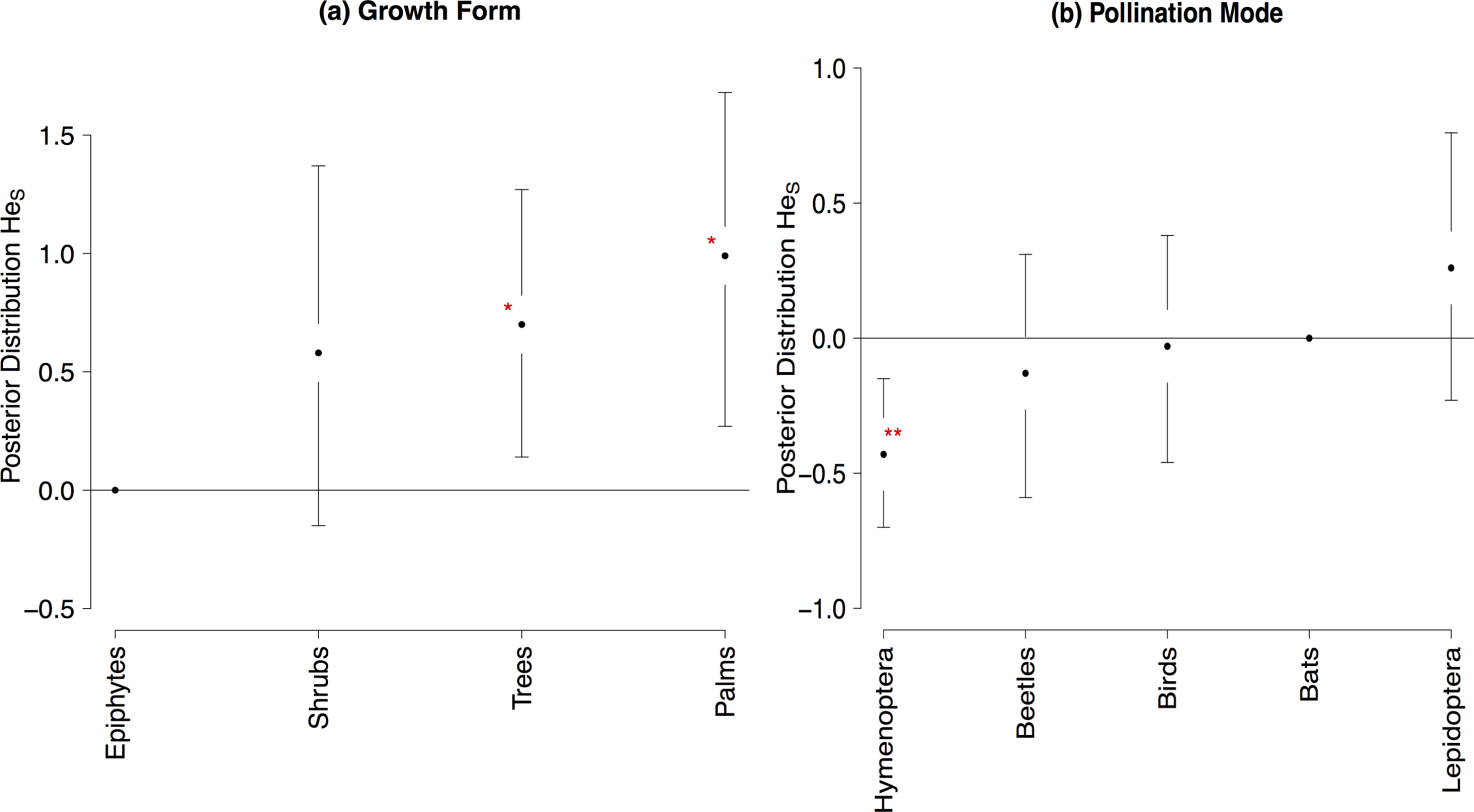
Mean values and 95% confidence intervals in the posterior distribution of the GLMM for genetic diversity overall populations (*He*_*S*_) for nuclear microsatellites. (**a**) Growth form (**b**) Habitat. Values highlighted by an asterisk are significant (* 0.05<P<0.01, ** P< 0.01).

Plants inhabiting rainforests showed slightly higher genetic diversity overall populations (*He*_*S*_, Fig 4A, S5 Table) for nuclear dominant markers. Furthermore, *He*_*S*_ values retrieved from nuclear microsatellite markers were higher in palms and trees than in epiphytes and shrubs, as well as in plants pollinated by Hymenoptera (Fig 5, S5 Table), while *He*_*S*_ retrieved from isozymes was not significantly related to any LHT or ecological attribute (S5 Table).

**Fig 6:**
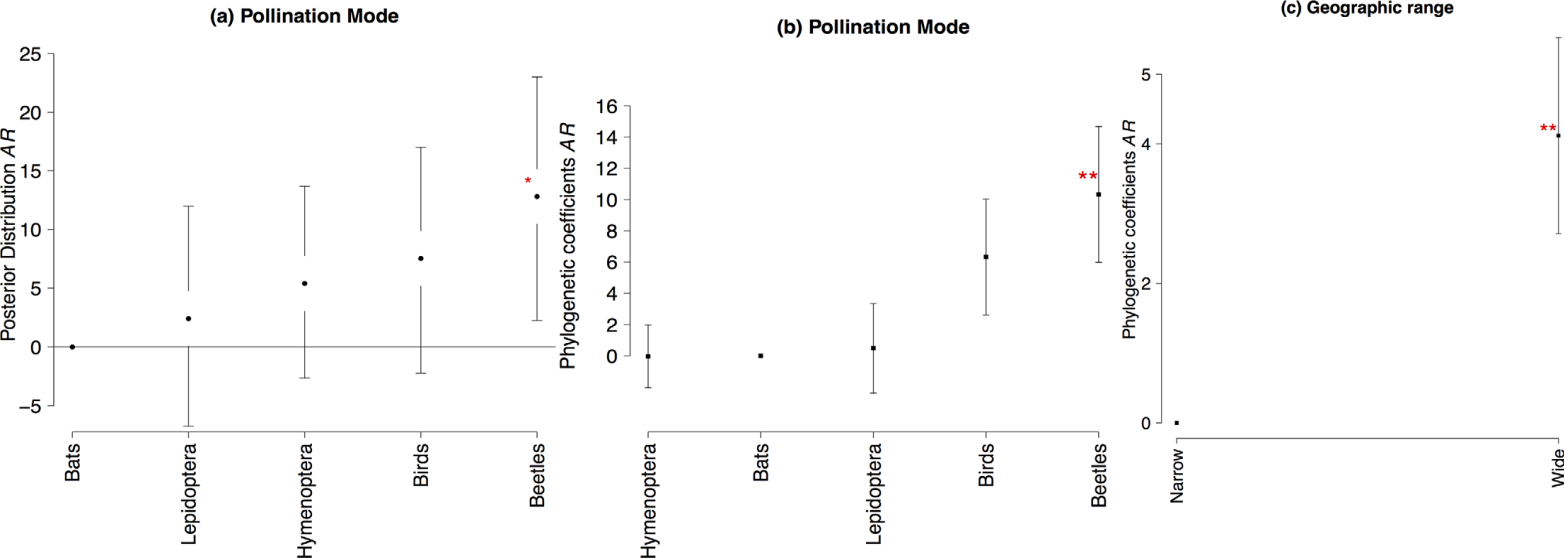
Mean values and 95% confidence intervals in the posterior distribution of the GLMM and phylogenetic coefficients and standard errors of pGLS for allelic richness (*AR*) for nuclear microsatellites. (**a**) Pollination mode (**b**) Pollination mode phylogenetic coefficients (**c**) Geographical range phylogenetic coefficients. Values highlighted by an asterisk are significant (* 0.05<P<0.01, ** P< 0.01).

Within population genetic diversity (*He*) was not significantly related to any LHT or ecological attribute studied (S6 Table), neither were haplotype (*h*) and nucleotide (π) diversities from nuclear and chloroplast sequences (S6 and S8 Tables), nor allelic richness retrieved from isozymes. Yet, allelic richness retrieved from nuclear microsatellites showed significant higher values for plants pollinated by beetles than for other pollination modes (Fig 6A, S6 Table).

#### Accounting for phylogenetic relationships in genetic patterns

**Fig 7:**
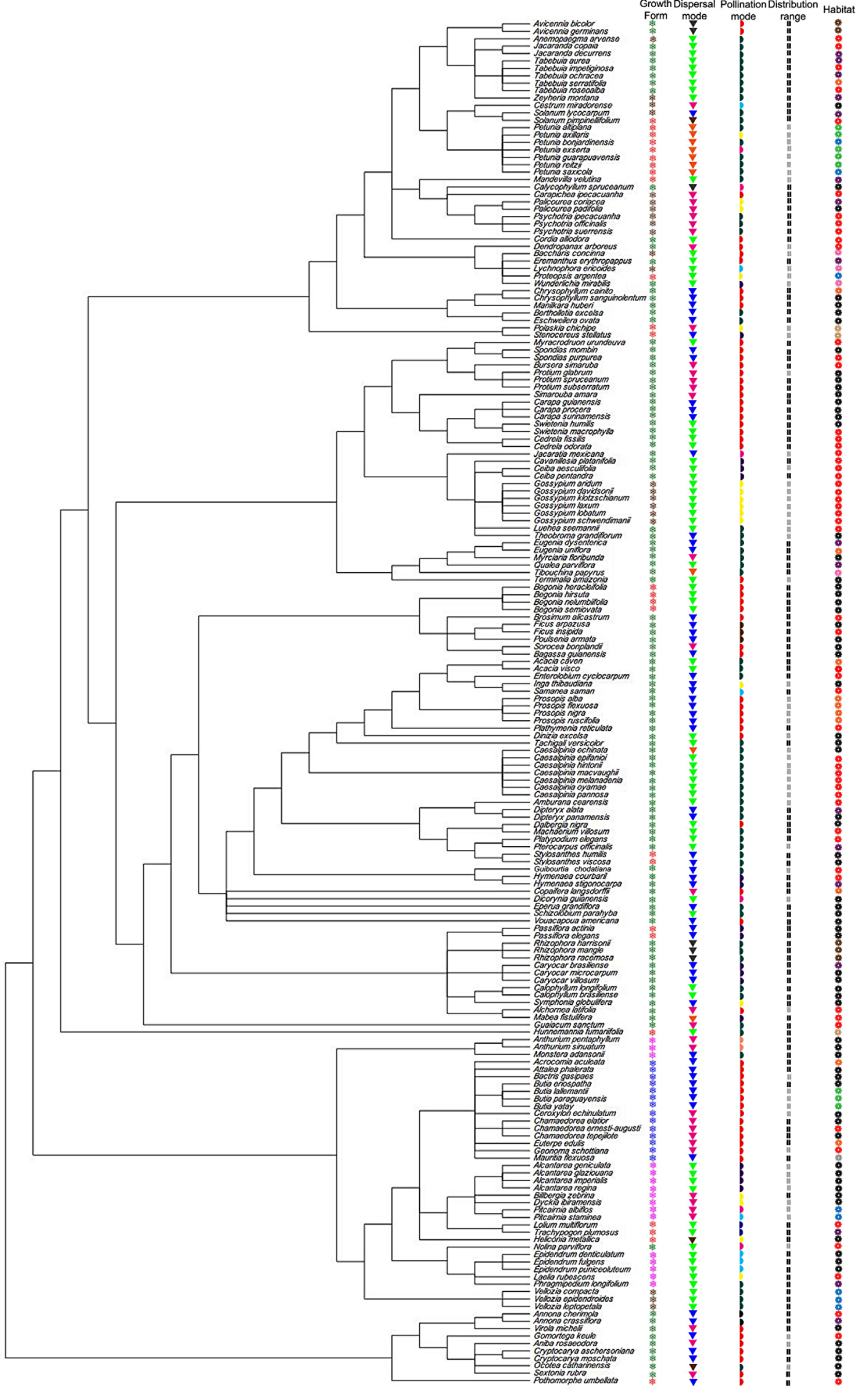
Phylogenetic super-tree of the Neotropical plants included in the analyses, obtained from Phylomatic using the internal master tree *Phylomatic tree R20120829*, with life-history traits and ecological attributes mapped. Growth form (❅): Fuchsia = Epiphytes, Red = Herbs, Blue = Palms, Brown = Shrubs, Green = Trees. Dispersal mode (▼): Orange = autochory, Brown = mixed (mammals and birds), Green = bats, Magenta = birds, Black = hydrochory, Dark blue = mammals, Light green = wind. Pollination mode (◗): Black = beetles, Yellow = birds, Brown = wasps, Red = small bees, Dark blue = wind, Purple = bats, Light blue = butterflies, Magenta = moth, Dark green = large bees, Light orange = flies. Geographical range (❙❙): Black = widespread, Grey = narrow. Habitat (❁): Sand = desert, Red = seasonally dry forests, Black = rainforests, Light green = grasslands, Brown = mangroves, Orange = mixed (rainforests and seasonally dry forests), Blue = rocky fields, Pink = rocky savannas, purple = savannas, Grey = wetlands. Reproductive system (❃): Brown = hermaphrodite, Dark blue = monoecious, Magenta = dioecious. Mating system (✭): Dark green = mixed, Dark red = outcrossing.

The test of serial independence showed that some LHT presented a significant phylogenetic signal (Table 4, see also Fig 7). For species with data for *He*_*S*_ for dominant markers, pollination mode, breeding system and geographical range had no significant phylogenetic signal (Table 4). For species with data for *AR* and *F*_*ST*_ for nuclear microsatellites, most LHT and ecological attributes had no significant phylogenetic signal (Table 4).

**Table 4:** Phylogenetic signal of life-history traits and ecological attributes for Neotropical plants included in the analyses of genetic diversity and differentiation using Abouheif’s proximity test of serial independence. Values followed by *ns* are not significant, p > 0.05. *F*_*ST*_, genetic differentiation among populations; *He*_*S*_, genetic diversity overall population; *F*_*IS*_, inbreeding coefficient; *AR*, allelic richness.

Life-history trait and ecological attribute	Observed Moran’s I	P-value
***F***_***ST***_ **(nuclear microsatellites)**		
**Growth form**	0.081 ^ns^	0.061
**Dispersal mode**	0.067 ^ns^	0.155
**Pollination mode**	0.066 ^ns^	0.161
**Breeding system**	0.060 ^ns^	0.212
**Mating system**	0.070 ^ns^	0.103
**Geographical range**	0.057 ^ns^	0.267
**Habitat**	0.045 ^ns^	0.481
***He***_***S***_ **(Dominant nuclear markers)**		
**Growth form**	0.495	0.001
**Dispersal mode**	0.209	0.053
**Pollination mode**	-0.061 ^ns^	0.455
**Breeding system**	0.139 ^ns^	0.100
**Mating system**	0.732	0.001
**Geographical range**	0.200 ^ns^	0.062
**Habitat**	0.321	0.016
***F***_***IS***_ **(Isozymes)**		
**Growth form**	0.167	0.053
**Dispersal mode**	-0.104 ^ns^	0.729
**Pollination mode**	-0.053 ^ns^	0.545
**Breeding system**	-0.044 ^ns^	0.488
**Mating system**	0.006 ^ns^	0.317
**Geographical range**	-0.092 ^ns^	0.673
**Habitat**	0.172 ^ns^	0.061
***AR* (nuclear microsatellites)**		
**Growth form**	0.341	0.004
**Dispersal mode**	-0.082 ^ns^	0.621
**Pollination mode**	0.000 ^ns^	0.318
**Breeding system**	-0.078 ^ns^	0.696
**Mating system**	-0.146 ^ns^	0.827
**Geographical range**	0.168	0.054
**Habitat**	-0.026 ^ns^	0.449
***F***_***ST***_ **(chloroplast)**		
**Growth form**	0.151	0.001
**Dispersal mode**	0.038 ^ns^	0.432
**Pollination mode**	0.057 ^ns^	0.108
**Breeding system**	0.041 ^ns^	0.312
**Mating system**	0.087	0.016
**Geographical range**	0.077	0.038
**Habitat**	0.072	0.040

Phylogenetic generalized least squares showed significant effects of pollination mode, mating system and habitat on genetic differentiation (*F*_*ST*_) for nuclear microsatellites (Fig 2E–2G, Tables 5 and 6). Plants pollinated by Lepidoptera had higher *F*_*ST*_ than plants pollinated by bats, beetles, birds and Hymenoptera insects (Fig 2E). In addition, plants with outcrossing mating system had significantly lower *F*_*ST*_ than mixed system species (Fig 2F). While, plants inhabiting mangroves, rocky fields and rocky savannas had higher *F*_*ST*_ than plants from other habitats (Fig 2G, Table 6).

**Table 5:** Phylogenetic generalized least squares for pollination mode and mating system, for each genetic parameter analyzed. Significant values are denoted in bold. *F*_*ST*_, genetic differentiation among populations; *F*_*IS*_, inbreeding coefficient; *Hes*, genetic diversity overall populations; *AR* allelic richness. SE, standard error.

	Parameter	F_ST_ (nuclear microsatellites)			*F*_*IS*_ (isozymes)			*He*_*S*_ (dominant nuclear markers)			*AR* (nuclear microsatellites)			*F*_*ST*_ (chloroplast markers)		
Life history trait	Variable	Coefficient±SE	T-value	P-value	Coefficient±SE	T-value	P-value	Coefficient±SE	T-value	P-value	Coefficient±SE	T-value	P-value	Coefficient±SE	T-value	P-value
**Pollination mode**	Intercept	-0.115±0.187	-0.616	0.542	0.067±0.288	0.234	0.817	0.279±0.229	1.219	0.249	4.004±3.638	3.638	1.101	0.519±0.290	1.786	0.081
	Bats	-	-	-	-	-	-	-	-	-	-	-	-	-	-	-
	Beetles	0.389±0.227	1.712	0.096	-0.066±0.334	-0.198	0.845	-	-	-	**10.326±4.357**	**2.370**	**0.026**	-	-	-
	Flies	-	-	-	-	-	-	-0.044±0.301	-0.145	0.888	-	-	-	-	-	-
	Hymenoptera	0.264±0.147	1.801	0.081	0.038±0.224	0.169	0.867	0.040±0.204	0.194	0.850	-0.033±2.015	-0.016	0.987	0.229±0.206	1.111	0.273
	Hummingbirds	0.048±0.168	0.287	0.776	0.303±0.270	1.120	0.273	-	-	-	6.318±3.705	1.705	0.101	0.273±0.259	1.053	0.298
	Lepidoptera	**0.414±0.171**	**2.424**	**0.021**	-0.174±0.255	-0.681	0.501	0.0070.256	0.029	0.978	0.483±2.870	0.168	0.868	0.172±0.242	0.711	0.481
	Wind	-	-	-	-	-	-	0.415±0.287	1.446	0.176	-	-	-	-	-	-
**Mating system**	Intercept	0.321±0.132	2.430	0.020	0.128±0.237	0.540	0.594	0.327±0.127	2.576	0.022	10.301±2.425	4.248	0.000	0.780±0.230	3.389	0.001
	Mixed	-	-	-	0.079±0.183	0.429	0.671	-	-	-	-	-	-	-0.138±0.171	-0.807	0.424
	Outcrossed	**-0.176±0.062**	**-2.843**	**0.007**	-0.058±0.165	-0.351	0.728	-0.087±0.125	-0.691	0.501	0.787±1.310	0.600	0.553	-0.140±0.156	-0.900	0.373

**Table 6:** Phylogenetic generalized least squares for ecological attributes (i.e. habitat and geographical range), for each genetic parameter analyzed. Significant values are denoted in bold. *F*_*ST*_, genetic differentiation among populations; *F*_*IS*_, inbreeding coefficient; *Hes*, genetic diversity overall populations; *AR* allelic richness; SE, standard error.

	Parameter	*F*_*ST*_ (nuclear microsatellites)			*F*_*IS*_ (isozymes)			*He*_*S*_ (dominant nuclear markers)			*AR* (nuclear microsatellites)			F_ST_(Chloroplast markers)		
Ecological attribute	Variable	Coefficient±SE	T-value	P-value	Coefficient±SE	T-value	P-value	Coefficient±SE	T-value	P-value	Coefficient±SE	T-value	P-value	Coefficient±SE	T-value	P-value
**Habitat**	Intercept	0.045±0.153	0.297	0.769	0.089±0.246	0.362	0.720	0.574±0.102	5.647	0.000	8.709±3.937	2.212	0.038		1.786	0.081
	Deserts	-	-	-	-	-	-	-	-	-	-	-	-	-	-	-
	Grasslands							-	-	-				-	-	-
	Mangroves	**0.473±0.150**	**3.157**	**0.004**	-	-	-				-	-	-	-	-	-
	Mixed	-0.155±0.176	-0.878	0.387				**-0.651±0.100**	**-6.479**	**0.000**		-				
	Rainforests	0.107±0.139	0.772	0.446	0.011±0.161	0.071	0.944	**-0.443±0.093**	**-4.762**	**0.001**						
	Rocky fields	**0.591±0.195**	**3.034**	**0.005**	0.207±0.249	0.828	0.415	**-0.450±0.126**	**-3.580**	**0.006**						
	Rocky savannas	**0.673±0.182**	**3.707**	**0.001**	-	-	-				-	-	-	-	-	-
	Savannas	0.226±0.142	1.588	0.122	-0.057±0.240	-0.237	0.814	**-0.298±0.120**	**-2.475**	**0.035**						
	SDTFs	0.032±0.140	0.224	0.824	-0.050±0.172	-0.292	0.773	-0.185±0.090	-2.059	0.070						
	Wetlands							**-0.580±0.123**	**-4.715**	**0.001**						
**Geographical range**	Intercept	0.202±0.139	1.453	0.155	0.117±0.184	0.633	0.532	0.478±0.158	3.023	0.009						
	Narrow	-	-	-				-	-	-	-	-	-		-	
	Widespread	-0.045±0.072	-0.635	0.529	-0.025±0.103	-0.248	0.806	-0.175±0.112	-1.559	0.141					-	

Likewise, habitat significantly affected genetic diversity overall populations (*He*_*S*_) for dominant markers (Fig 4B). *He*_*S*_ values were significantly lower for plants inhabiting mixed, rainforest rocky fields, savannas and wetlands (Table 6).

Allelic richness for nuclear microsatellites had significantly higher values for plants pollinated by beetles than other pollination modes (Fig 6B, Table 5) and plants with wide geographical range than narrow (Fig 6C, Table 6). However growth form (S9 Table), dispersal mode (S10 Table) and breeding system (S11 Table) had no significant effects on AR when phylogenetic relationships where accounted for. We also found no significant effects of LHT nor ecological attributes on *F*_*IS*_ for isozymes, *H*_*es*_ for microsatellites and *F*_*ST*_ for chloroplast markers when phylogenetic relationships were accounted for.

## Discussion

### General genetic patterns

Plants pollinated by Hymenoptera have higher *F*_*ST*_ and *He*_*S*_ for nuclear microsatellites. Although bees may fly long distances and may potentially promote long distance pollen dispersal they may also display temporary specialization and stay in the same plant patch [42] leading to low gene flow. However, many works using parentage analysis showed long-distance pollen dispersal in Neotropical trees [14]. Though, the higher *F*_*ST*_ and *He*_*S*_ for plants pollinated by Hymenoptera may be an artifact of phylogenetic signal since it disappears when accounting for phylogenetic independence (see below). Pollination mode was also correlated to allelic richness for nuclear microsatellites. We found data on *AR* for only one species pollinated by beetles (*Annona crassiflora*, Annonaceae). *Annona* species are protogynous [43], which potentially promote long-distance pollen flow increasing allelic richness. Yet, this result may also be an artifact of taxonomic sampling bias due to the low number of species and biased family sampling. Notwithstanding, this relationship was maintained when phylogenetic non-independence was accounted for (see below).

Dispersal mode and breeding system explained differences in genetic differentiation at nuclear microsatellite markers, but the relationships were not recovered when phylogenetic non-independence was accounted for (see below). Wind- and mammal-dispersed species had lower genetic differentiation at nuclear genome. Similar to large sized mammals, wind may promote long distance seed dispersal [44], potentially increasing gene flow and decreasing genetic differentiation among population. Dioecious species had lower *F*_*ST*_ at nuclear microsatellites than monoecious and hermaphrodite species. Dioecious species are obligate outcrossing which is correlated to long-distance gene flow [45], decreasing population genetic differentiation. In addition, all dioecious species studied so far in Neotropics are long-lived trees or palms, which also presented lower genetic differentiation. The association between dioecy and woodiness may be an outcome of strong selection for outcrossing in plants with a long life span [46].

Mating or breeding systems did not explained variation in *F*_*IS*_, but growth form and habitat did. Growth form was an important LHT in predicting genetic differentiation at chloroplast genome, inbreeding for isozymes and genetic diversity overall populations for nuclear microsatellites in Neotropical plants. Herbs showed a higher *F*_*IS*_ value than other growth forms, it might be due to a sampling effect since we found data on inbreeding only for three herb species (one Poaceae species and two Begoniaceae). For chloroplast genome, shrubs presented higher genetic differentiation than the other life forms. In fact, we found data for chloroplast genome for seven shrub species (six Malvaceae and one Rubiaceae) from different habitats (rainforests, SDTF and rocky savannas), with different pollination (bees, birds, butterflies) and dispersal modes (birds, wind and mammals). Many Malvaceae species have mixed-mating system with outcrossing rates lower than 80%, and also apomictic seed production (see [32,47] for a review) that may decrease genetic diversity within population and increase genetic differentiation among populations.

Although plants from rocky fields showed significantly higher inbreeding coefficients (*F*_*IS*_), than other habitats, this result may be the effect of taxonomic sampling bias (i.e. low number of species and biased family sampling in Asteraceae (1) and Veloziaceae (2)). In addition, mean posterior value of *F*_*IS*_ for rocky field plants was only slightly higher than zero (see Fig 2B) and the effect was not recovered when phylogenetic dependence was taken into account (see below). Genetic diversity overall populations (*He*_*S*_) was slightly higher for plants from rainforests for nuclear dominant markers. Due to patterns in seed mortality and spatial recruitment patterns of trees (see [48] for a review], gene flow may reach long distances in rainforests increasing effective population size and genetic diversity [10].

### Genetic patterns accounting for phylogenetic non-independence

As expected, the non-independence of LHTs along sister clades (i.e. related species have more similar LHT than expected by chance) had significant effects on the genetic patterns detected in Neotropical plants. When accounted for phylogenetic non-independence in nuclear microsatellites, pollination mode, mating system and habitat had significant effects on genetic differentiation (*F*_*ST*_) whereas pollination mode and geographical range had a significant effect on allelic richness (*AR*). Species pollinated by Lepidoptera (butterflies and moths) had higher genetic differentiation than the other pollination modes. Butterflies and moth usually fly short distances (see [49] and references therein) leading to short distance pollen flow increasing genetic differentiation among populations. In addition, the higher allelic richness for species pollinated by beetles was recovered when accounted for phylogenetic non-independence. The relationships of all other LHT (e.g. growth form, breeding system and dispersal mode) on genetic differentiation found by GLMM were not recovered, although these LHT had no significant phylogenetic signal for species analyzed for nuclear microsatellites. For chloroplast *F*_*ST*_, the effect of growth form showed by the GLMM was most likely due to phylogenetic signal because no significant effect was detected when phylogenetic non-independence was taken into account.

Habitat was the only ecological attribute showing significant effects on genetic diversity overall population (*Hes*) for dominant nuclear markers. Species occurring in rainforest, rocky fields, savannas, wetlands and mixed forests had significant lower values of genetic differentiation than grasslands and SDTFs.

Our findings showed that different results might be retrieved when phylogenetic non-independence is taken into account. However, the surveyed literature was biased for most LHT and attributes, mainly due to the low number of genera (116) and families (45) studied so far in the Neotropics. For instance, all epiphytes are from three families (Araceae, Bromeliaceae, Orchidaceae). For Orchidaceae, only three genera were studied and for Araceae only two genera. For pollination mode, for instance, all wasp-pollinated species are Moraceae (*Ficus* spp.) and all fly pollinated species are Araceae. Thus, the analysis of pattern of genetic diversity and differentiation in relation to LHT and plant attributes may require larger samples sizes, i.e. large taxonomic sampling and also wide variation in LHTs and ecological attributes. Such information is unfortunately lacking so far and efforts should be guided in this direction in the future.

Moreover, the detection of phylogenetic signal for a given trait depends on the sampling (taxon sampling, number of species) and accuracy of phylogenetic tree [24,50]. Thus, although we found no significant phylogenetic signal for some traits, for instance, dispersal mode and breeding system, it does not mean that the trait has not evolved under a Brownian motion model and rather than a taxonomic sampling bias is what may not allow us the detection of any pattern of phylogenetic signal. It is important to note that traits related to seed dispersal mode (e.g. seed size and number) in Angiospermae have significant phylogenetic signal and evolutionary constrains imposed by reproductive structures [51]. Also, many traits in seed plants have evolutionary interdependencies that may constrain evolutionary and adaptive responses [52]. Actually, the Neotropical plants studied so far showed a significant relationship of pollination mode and mating system with genetic differentiation, yet showed no significant phylogenetic signal in our data.

We found no effect of dispersal mode on genetic differentiation at nuclear microsatellites, despite the non-significant phylogenetic signal in our data. Our findings agree with previous predictions that the general higher rates of pollen flow compared to seed flow could obscure the effects of seed dispersal mode on genetic differentiation at nuclear molecular markers [17,53]. Indeed, many works show higher contribution of pollen dispersal than seed dispersal to gene flow in Angiospermae [53] and in Neotropical plants [54,55]. In the surveyed data, we found genetic differentiation for both chloroplast (*FST*_*m*_) and nuclear genome (*FST*_*b*_) for 16 species. The ratio of pollen to seed flow considering inbreeding calculated as proposed by [56], i.e. (pollen flow)/(seed flow) = {[(1/*F*_*STb*_) - 1] x (1 + *F*_*IS*_) - 2[(1/*F*_*STm*_) - 1]}/[(1/*F*_*STm*_) - 1], was higher than 1 for all species except one, indicating higher contribution of pollen than seed to historical gene flow, which may explains our non significant results.

Despite the differences in data set, our results agree with that obtained by [17]. They also found no significant relationships of breeding system for genetic differentiation for both nuclear molecular markers and maternally inherited markers but significant effects for mating system and pollination mode for nuclear molecular markers. They analyzed 150 species from all climate zones worldwide but species from Northern Hemisphere are overrepresented in their data. Here, we focused exclusively on Neotropical species and analyzed a rather larger data set (186 species). Thus, phylogenetic relationships among analyzed species may be different due to taxonomic and evolutionary history in different climatic zones and biomes. However, our finds on the effects of dispersal mode differ from [17].

Many works found relationships between growth form and genetic polymorphism and differentiation for allozymes/isozymes, i.e. long-lived woody perennials have higher levels of allozyme variation and lower genetic differentiation [6–9]. Our results do not corroborate this pattern. We found significant relationships for isozymes only between inbreeding coefficient and growth form and habitat. Though such relationships did not hold anymore when phylogenetic non-independence was taken into account, most likely due to the phylogenetic signal in growth form and also due to the different taxonomic sampling bias (their data included Gymnospermae and more species from Temperate than Neotropical regions).

## Concluding Remarks

Our findings show that LHTs affect substantially the pattern of genetic diversity in Neotropical plants, hence may drive key evolutionary processes along with ecological features. Considering phylogenetic non-independence, pollination mode and mating system were the LHT with significant effects on genetic differentiation (*F*_*ST*_) and pollination mode for allelic richness (*AR*) for nuclear microsatellites. Ecological attributes also explained genetic differentiation for nuclear microsatellites. For chloroplast genome, studies published so far using CAPs, sequencing or microsatellites show no significant patterns associated to LHT or ecological attributes in genetic differentiation neither in genetic diversity (*h* and π). Genetic diversity for nuclear genome (*He*_*S*_ and *He*) could not be explained by any LHT or geographical range. However, habitat was significantly related to genetic diversity overall population (*He*_*S*_) at nuclear dominant markers. We expected that trees have significant higher genetic diversity and allelic richness, and lower *F*_*ST*_ and *F*_*IS*_ than the other growth forms. However, we found no significant effect of growth form in any genetic parameter analyzed. Nevertheless, plants with outcrossing mating system had lower *F*_*ST*_.

Since here we showed that some LHT are phylogenetically non-independent and that the relationship between LHT and ecological attributes and genetic parameters might be different when phylogenetic non-independence is taken into account, we call for caution when interpreting results from previous reviews. Most reviews seeking for patterns in genetic diversity and differentiation underestimated (or even did not account for) the effect of phylogenetic non-independence. Spurious relationships may thus arise from conventional statistical analysis by inflating type I error when not accounted for such non-independence. We acknowledge the limitations of our results due to biased taxonomical sampling in the data set and the low number of studied species for some LHT. However, this is a first attempt to find patterns in genetic diversity and genetic differentiation for Neotropical plants using a large data set from Neotropical plants.

## Supporting Information

S1 Fig Mean values and 95% confidence intervals of the posterior distribution of the GLMM for genetic differentiation among populations for chloroplast genome. (TIFF)

S1 Table Number of species per life-history trait (LHT) across all the studies included in the analyses of genetic diversity and structure in Neotropical plants. (DOCX)

S2 Table Number of species per ecological attributes across all the studies included in the analyses of genetic diversity and structure in Neotropical plants. (DOCX)

S3 Table Number of species analyzed per molecular marker across all the studies included in the analyses of genetic diversity and structure in Neotropical plants. (DOCX)

S4 Table Number of genus and species per family included in the analyses of genetic diversity and structure in Neotropical plants. (DOCX)

S5 Table Mean values of the posterior distribution of the GLMM for nuclear genome, for genetic parameters. *F*_*ST*_, genetic differentiation among populations; *He*_*S*_, mean genetic diversity among populations. Note that both genetic parameters are calculated for dominant (i.e. AFLP, RAPD,ISSR.), isozymes and microsatellite makers. Significant values are denoted in bold and grey-shaded.
(DOCX)

S6 Table Mean values of the posterior distribution of the GLMM for nuclear genome, for genetic parameters. *He*, genetic diversity within population; *F*_*IS*_, inbreeding coefficient; *AR*, allelic richness. Note that both genetic parameters are calculated for isozymes and microsatellite makers. Significant values are denoted in bold and grey-shaded.
(DOCX)

S7 Table Mean values of the posterior distribution of the GLMM for nuclear genome, for genetic parameters. *h*, haplotype diversity π, nucleotide diversity. Significant values are denoted in bold.
(DOCX)

S8 Table Mean values of the posterior distribution of the GLMM for chloroplast genome, for genetic parameters. *F*_*ST*_, genetic differentiation among populations; *h*, haplotype diversity; π, nucleotide diversity. Significant values are denoted in bold and grey-shaded.
(DOCX)

S9 Table Phylogenetic generalized least squares for growth form, for each genetic parameter analyzed. Significant values are denoted in bold. *F*_*ST*_, genetic differentiation among populations; *He*_*S*_, mean genetic diversity among populations, *He*, genetic diversity within population; *F*_*IS*_, inbreeding coefficient. SE, standard error.
(DOCX)

S10 Table Phylogenetic generalized least squares for dispersal mode, for each genetic parameter analyzed. Significant values are denoted in bold. *F*_*ST*_, genetic differentiation among populations; *He*_*S*_, mean genetic diversity among populations, *He*, genetic diversity within population; *F*_*IS*_, inbreeding coefficient. SE, standard error.
(DOCX)

S11 Table Phylogenetic generalized least squares for breeding system, for each genetic parameter analyzed. Significant values are denoted in bold. *F*_*ST*_, genetic differentiation among populations; *He*_*S*_, mean genetic diversity among populations, *He*, genetic diversity within population; *F*_*IS*_, inbreeding coefficient. SE, standard error.
(DOCX)
