# Correction: Pollination Mode and Mating System Explain Patterns in Genetic Differentiation in Neotropical Plants

**DOI:** 10.1371/journal.pone.0184674

**Published:** 2017-09-07

**Authors:** Liliana Ballesteros-Mejia, Natácia E. Lima, Matheus S. Lima-Ribeiro, Rosane G. Collevatti

The image for Fig 7 is missing columns “Reproductive system” and “Mating system.” Please see the corrected Fig 7 below.

**Fig 7:**
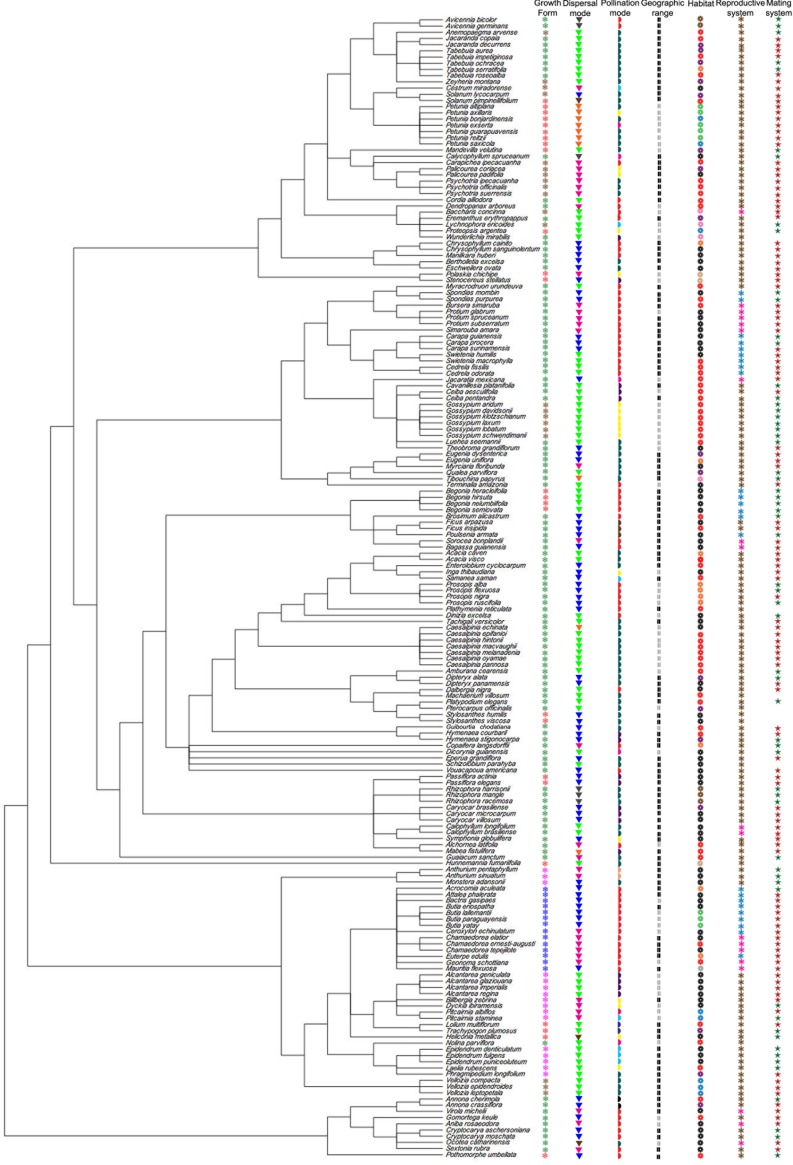
Phylogenetic super-tree of the Neotropical plants included in the analyses, obtained from Phylomatic using the internal master tree *Phylomatic tree R20120829*, with life-history traits and ecological attributes mapped. Growth form (❅): Fuchsia = Epiphytes, Red = Herbs, Blue = Palms, Brown = Shrubs, Green = Trees. Dispersal mode (▼): Orange = autochory, Brown = mixed (mammals and birds), Green = bats, Magenta = birds, Black = hydrochory, Dark blue = mammals, Light green = wind. Pollination mode (◗): Black = beetles, Yellow = birds, Brown = wasps, Red = small bees, Dark blue = wind, Purple = bats, Light blue = butterflies, Magenta = moth, Dark green = large bees, Light orange = flies. Geographical range (❙❙): Black = widespread, Grey = narrow. Habitat (❁): Sand = desert, Red = seasonally dry forests, Black = rainforests, Light green = grasslands, Brown = mangroves, Orange = mixed (rainforests and seasonally dry forests), Blue = rocky fields, Pink = rocky savannas, purple = savannas, Grey = wetlands. Reproductive system (❃): Brown = hermaphrodite, Dark blue = monoecious, Magenta = dioecious. Mating system (✭): Dark green = mixed, Dark red = outcrossing.
