# Supplementary material for: Pollination Mode and Mating System Explain Patterns in Genetic Differentiation in Neotropical Plants
Source: PLoS One. 2016 Jul 29;11(7):e0158660. doi: 10.1371/journal.pone.0158660 (PMC4966973; doi:10.1371/journal.pone.0158660)
Supplement: S1 Table — (DOCX) [file pone.0158660.s002.docx]

**Pollination mode and mating system explains patterns in genetic diversity and differentiation in Neotropical plants**

Liliana Ballesteros-Mejia*^1^*, Natácia E Lima*^1^*, Matheus S. Lima-Ribeiro*^2^*, Rosane G Collevatti*^1^*

**S1 Table. Number of species per life-history trait (LHT) across all the studies included in the analyses of genetic diversity and structure in Neotropical plants.**

| **Life-history trait (LHT)** |  | **Number of species** |
| --- | --- | --- |
| **Growth form** | **Epiphyte** | 17 |
|  | **Herb** | 25 |
|  | **Palm** | 14 |
|  | **Shrub** | 21 |
|  | **Tree** | 109 |
| **Dispersal Mode** | **Bats** | 1 |
|  | **Birds** | 34 |
|  | **Hidrochory** | 7 |
|  | **Mammals** | 60 |
|  | **Mixed (birds and mammals)** | 3 |
|  | **Wind** | 81 |
|  | **Wind** | 71 |
|  | **Authochory** | 9 |
| **Pollination mode** | **Bats** | 18 |
|  | **Beetles** | 2 |
|  | **Lepidoptera** | 14 |
|  | **Butterflies** | 8 |
|  | **Moths** | 6 |
|  | **Flies** | 2 |
|  | **Hummingbirds** | 18 |
|  | **Hymenoptera** | 130 |
|  | **Large bees** | 63 |
|  | **Small bees** | 64 |
|  | **Wasps** | 3 |
|  | **Wind** | 2 |
| **Mating system** | **Mixed** | 62 |
|  | **Outcrossing** | 124 |
| **Breeding system** | **Dioecious** | 19 |
|  | **Monoecious** | 25 |
|  | **Hermaphrodite** | 142 |
