# Supplementary material for: Pollination Mode and Mating System Explain Patterns in Genetic Differentiation in Neotropical Plants
Source: PLoS One. 2016 Jul 29;11(7):e0158660. doi: 10.1371/journal.pone.0158660 (PMC4966973; doi:10.1371/journal.pone.0158660)
Supplement: S2 Table — (DOCX) [file pone.0158660.s003.docx]

**Pollination mode and mating system explains patterns in genetic diversity and differentiation in Neotropical plants**

Liliana Ballesteros-Mejia*^1^*, Natácia E Lima*^1^*, Matheus S. Lima-Ribeiro*^2^*, Rosane G Collevatti*^1^*

**S2 Table. Number of species per ecological attributes across all the studies included in the analyses of genetic diversity and structure in Neotropical plants.**

| **Ecological Attribute** |  | **Number of Species** |
| --- | --- | --- |
| **Habitat** | **Deserts** | 3 |
|  | **Grasslands** | 8 |
|  | **Mangroves** | 6 |
|  | **Mixed (rain forests and seasonally dry forests)** | 10 |
|  | **Rain forests** | 71 |
|  | **Rocky fields** | 8 |
|  | **Rocky savannas** | 4 |
|  | **Savannas** | 16 |
|  | **Seasonally dry forests** | 57 |
|  | **Wetlands** | 3 |
| **Geographic range** | **Narrow** | 71 |
|  | **Widespread** | 115 |
