# Supplementary material for: Pollination Mode and Mating System Explain Patterns in Genetic Differentiation in Neotropical Plants
Source: PLoS One. 2016 Jul 29;11(7):e0158660. doi: 10.1371/journal.pone.0158660 (PMC4966973; doi:10.1371/journal.pone.0158660)
Supplement: S3 Table — (DOCX) [file pone.0158660.s004.docx]

**Pollination mode and mating system explains patterns in genetic diversity and differentiation in Neotropical plants**

Liliana Ballesteros-Mejia*^1^*, Natácia E Lima*^1^*, Matheus S. Lima-Ribeiro*^2^*, Rosane G Collevatti*^1^*

**S3 Table. Number of species analyzed per molecular marker across all the studies included in the analyses of genetic diversity and structure in Neotropical plants.**

| **Molecular Marker** | **Number of Species** |
| --- | --- |
| **Dominant Markers** | 72 |
| **AFLP** | 29 |
| **RAPD** | 38 |
| **ISSR** | 5 |
| **Co-dominant Markers** | 112 |
| **Isozyme/allozyme** | 51 |
| **Nuclear microsatellite** | 61 |
| **CAPs** | 11 |
| **Chloroplast microsatellite** | 17 |
| **Chloroplast sequence** | 51 |
| **Nuclear sequence** | 24 |
