# Supplementary material for: Pollination Mode and Mating System Explain Patterns in Genetic Differentiation in Neotropical Plants
Source: PLoS One. 2016 Jul 29;11(7):e0158660. doi: 10.1371/journal.pone.0158660 (PMC4966973; doi:10.1371/journal.pone.0158660)
Supplement: S4 Table — (DOCX) [file pone.0158660.s005.docx]

**Pollination mode and mating system explains patterns in genetic diversity and differentiation in Neotropical plants**

Liliana Ballesteros-Mejia*^1^*, Natácia E Lima*^1^*, Matheus S. Lima-Ribeiro*^2^*, Rosane G Collevatti*^1^*

**S4 Table. Number of genus and species per family included in the analyses of genetic diversity and structure in Neotropical plants.**

| Family | Genus | Number of Species |
| --- | --- | --- |
| Acanthaceae | 1 | 2 |
| Anacardiaceae | 2 | 3 |
| Annonaceae | 1 | 2 |
| Apocynaceae | 1 | 1 |
| Araceae | 2 | 3 |
| Araliaceae | 1 | 1 |
| Arecaceae | 9 | 14 |
| Asparagaceae | 1 | 1 |
| Asteraceae | 5 | 5 |
| Begoniaceae | 1 | 4 |
| Bignoniaceae | 4 | 9 |
| Boraginaceae | 1 | 1 |
| Bromeliaceae | 4 | 8 |
| Burseraceae | 2 | 4 |
| Cactaceae | 2 | 2 |
| Calophyllaceae | 1 | 1 |
| Caricaceae | 1 | 1 |
| Caryocaraceae | 1 | 4 |
| Clusiaceae | 1 | 1 |
| Combretaceae | 1 | 1 |
| Euphorbiaceae | 2 | 2 |
| Fabaceae | 23 | 36 |
| Gomortegaceae | 1 | 1 |
| Heliconiaceae | 1 | 1 |
| Lauraceae | 4 | 5 |
| Lecythidaceae | 2 | 2 |
| Malvaceae | 5 | 11 |
| Melastomataceae | 1 | 1 |
| Meliaceae | 3 | 7 |
| Moraceae | 5 | 6 |
| Myristicaceae | 1 | 1 |
| Myrtaceae | 2 | 3 |
| Orchidaceae | 4 | 6 |
| Papaveraceae | 1 | 1 |
| Passifloraceae | 1 | 2 |
| Pellicieraceae | 1 | 1 |
| Piperaceae | 1 | 1 |
| Poaceae | 2 | 2 |
| Rhizophoraceae | 1 | 3 |
| Rubiaceae | 4 | 7 |
| Sapotaceae | 2 | 3 |
| Simaroubaceae | 1 | 1 |
| Solanaceae | 3 | 10 |
| Velloziaceae | 1 | 3 |
| Vochysiaceae | 1 | 1 |
| Zygophyllaceae | 1 | 1 |
| Total | **116** | **186** |
