# Supplementary material for: Pollination Mode and Mating System Explain Patterns in Genetic Differentiation in Neotropical Plants
Source: PLoS One. 2016 Jul 29;11(7):e0158660. doi: 10.1371/journal.pone.0158660 (PMC4966973; doi:10.1371/journal.pone.0158660)
Supplement: S5 Table — FST, genetic differentiation among populations; HeS, mean genetic diversity among populations. Note that both genetic parameters are calculated for dominant (i.e. AFLP, RAPD,ISSR.), isozymes and microsatellite makers. Significant values are denoted in bold and grey-shaded. (DOCX) [file pone.0158660.s006.docx]

**Pollination mode and mating system explains patterns in genetic diversity and differentiation in Neotropical plants**

Liliana Ballesteros-Mejia*^1^*, Natácia E Lima*^1^*, Matheus S. Lima-Ribeiro*^2^*, Rosane G Collevatti*^1^*

**S5 Table.** **Mean values of the posterior distribution of the GLMM for nuclear genome, for genetic parameters.** *F_ST_*, genetic differentiation among populations; *He_S_*, mean genetic diversity among populations. Note that both genetic parameters are calculated for dominant (i.e. AFLP, RAPD,ISSR.), isozymes and microsatellite makers. Significant values are denoted in bold and grey-shaded.

| Class  Parameter | | *Dominant* | | *Isozymes* | | *Microsatellites* | | *Dominant* | | *Isozymes* | | *Microsatellites* | |
| --- | --- | --- | --- | --- | --- | --- | --- | --- | --- | --- | --- | --- | --- |
|  |  | ***F_ST_*** | | ***F_ST_*** | | ***F_ST_*** | | ***H_S_*** | | ***H_S_*** | | ***H_S_*** | |
|  |  | **Mean** | **P-value** | **Mean** | **P-value** | **Mean** | **P-value** | **Mean** | **P-value** | **Mean** | **P-value** | **Mean** | **P-value** |
| Growth form | **Herb** | -0.377 | 0.241 | 763.80 | 1 | 347.80 | 0.997 | -278.80 | 0.993 | -0.60 | 0.183 | -237.1 | 0.990 |
|  | **Palm** | 0.176 | 0.281 | -681.50 | 0.984 | **-0.56** | **0.004** | -462.30 | 0.999 | 584 | 0.984 | **0.99** | **0.016** |
|  | **Shrub** | 0.072 | 0.663 | -681.40 | 0.984 |  |  | -1299.00 | 0.994 | 584 | 0.984 | 0.58 | 0.124 |
|  | **Tree** | 0.231 | 0.159 | -681.50 | 0.984 | **-0.39** | **0.009** | -1030.00 | 0.990 | 584 | 0.984 | **0.70** | **0.025** |
| Dispersal Mode | **Birds** | -0.363 | 0.283 |  |  |  |  |  |  | - | - |  |  |
|  | **Hidrochory** | -0.073 | 0.420 |  |  | 975.50 | 0.988 | 43.65 | 0.992 |  |  | 1137 | 0.993 |
|  | **Mammals** | -0.209 | 0.375 | -0.039 | 0.764 | **-0.55** | **0.000** | -838.00 | 0.976 | 0.20 | 0.384 | -0.07 | 0.846 |
|  | **Mixed** | -0.090 | 0.366 | -0.040 | 0.831 |  |  |  |  | 0.14 | 0.610 |  |  |
|  | **Wind** |  |  | -0.062 | 0.590 | **-0.74** | **0.000** | -163.60 | 0.987 | -0.24 | 0.191 | 0.21 | 0.532 |
| Pollination Mode | **Beetles** |  |  | 0.0135 | 0.930 | 0.15 | 0.204 |  |  | -0.44 | 0.058 | -0.13 | 0.535 |
|  | **Flies** | 0.094 | 0.705 |  |  |  |  | -865.60 | 0.980 |  |  |  |  |
|  | **Himenoptera** | 0.148 | 0.359 | -0.135 | 0.421 | **0.17** | **0.015** | -27.46 | 1.000 | 296.5 | 0.983 | **-0.43** | **0.006** |
|  | **Hummingbirds** | 0.030 | 0.890 | -681.50 | 0.984 | 0.10 | 0.371 | 121.00 | 0.996 | 881.0 | 0.981 | -0.03 | 0.875 |
|  | **Lepidoptera** | 0.302 | 0.232 | -0.293 | 0.237 | -0.05 | 0.659 | -980.10 | 0.996 | 297.0 | 0.983 | 0.26 | 0.301 |
|  | **Wind** | -0.042 | 0.862 | -1445.0 | 0.990 |  |  |  |  |  |  |  |  |
| Mating system | **Mixed** | 0.197 | 0.083 | 0.04 | 0.797 |  |  | 71.79 | 0.998 | -0.29 | 0.267 |  |  |
|  | **Outcrossing** | 0.150 | 0.182 | -0.07 | 0.608 | -0.07 | 0.167 | 254.30 | 0.999 | -0.22 | 0.239 | 0.13 | 0.270 |
| Breeding system | **Dioecious** |  |  | -0.04 | 0.857 |  |  |  |  | 75.4 | 0.994 |  |  |
|  | **Monoecious** | 0.139 | 0.273 | -0.07 | 0.75 | **0.63** | **0.001** | 183.80 | 0.994 | 0.27 | 0.361 | 0.01 | 0.967 |
|  | **Hermaphrodite** | 0.164 | 0.194 | 0.07 | 0.748 | **0.56** | **0.001** |  |  | 75.3 | 0.994 | 0.03 | 0.923 |
| Geographic range | **Wide** | 0.088 | 0.141 | -0.089 | 0.413 | 0.11 | 0.068 | -96.15 | 0.993 | 0.14 | 0.546 | 0.01 | 0.967 |
| Habitat | **Grasslands** |  |  | - | - | - | - | - | - | - | - | - | - |
|  | **Mangroves** | 0.455 | 0.252 | - | - | -627.10 | 0.997 | 96.09 | 0.998 | - | - | -1375 | 0.988 |
|  | **Mixed** | 0.174 | 0.218 | - | - | 348.20 | 0.997 | -742.50 | 0.984 | - | - | -237.5 | 0.990 |
|  | **Rain forests** | -0.007 | 0.954 | 0.02 | 0.851 | 348.80 | 0.997 | **-0.50** | **0.010** | -75.1 | 0.994 | -237.6 | 0.990 |
|  | **Rocky fields** | 0.332 | 0.106 | -0.16 | 0.499 | 348.60 | 0.997 | 345.80 | 0.996 | -74.4 | 0.994 | -237.5 | 0.990 |
|  | **Rocky savannas** | 0.185 | 0.381 |  |  | 349.50 | 0.997 |  |  |  |  | -237.8 | 0.990 |
|  | **Savannas** | -0.046 | 0.764 | -0.14 | 0.612 | 348.80 | 0.997 | 250.90 | 0.998 | 221.7 | 0.974 | -237.5 | 0.990 |
|  | **Seasonally dry forests** | 0.042 | 0.765 | -0.10 | 0.338 | 348.80 | 0.997 | 278.50 | 0.993 | -74.9 | 0.994 | -237.5 | 0.990 |
|  | **Wetlands** | 0.167 | 0.290 | 0.02 | 0.851 | - | - | -857.40 | 0.988 | - | - | - | - |
