# Supplementary material for: Pollination Mode and Mating System Explain Patterns in Genetic Differentiation in Neotropical Plants
Source: PLoS One. 2016 Jul 29;11(7):e0158660. doi: 10.1371/journal.pone.0158660 (PMC4966973; doi:10.1371/journal.pone.0158660)
Supplement: S6 Table — He, genetic diversity within population; FIS, inbreeding coefficient; AR, allelic richness. Note that both genetic parameters are calculated for isozymes and microsatellite makers. Significant values are denoted in bold and grey-shaded. (DOCX) [file pone.0158660.s007.docx]

**Pollination mode and mating system explains patterns in genetic diversity and differentiation in Neotropical plants**

Liliana Ballesteros-Mejia*^1^*, Natácia E Lima*^1^*, Matheus S. Lima-Ribeiro*^2^*, Rosane G Collevatti*^1^*

**S6 Table.** **Mean values of the posterior distribution of the GLMM for nuclear genome, for genetic parameters**. *He*, genetic diversity within population; *F_IS_*, inbreeding coefficient; *AR*, allelic richness. Note that both genetic parameters are calculated for isozymes and microsatellite makers. Significant values are denoted in bold and grey-shaded.

| Class  Parameter | | *Isozymes* | | *Microsatellites* | | *Isozymes* | | *Microsatellites* | | *Isozymes* | | *Microsatellites* | |
| --- | --- | --- | --- | --- | --- | --- | --- | --- | --- | --- | --- | --- | --- |
|  |  | *He* | | *He* | | *F_IS_* | | *F_IS_* | | *AR* | | *AR* | |
|  |  | **Mean** | **P-value** | **Mean** | **P-value** | **Mean** | **P-value** | **Mean** | **P-value** | **Mean** | **P-value** | **Mean** | **P-value** |
| Growth form | **Herb** | **-** | **-** |  |  | **-1.16** | **0.033** | -288.60 | 0.995 |  |  | -91.85 | 0.996 |
|  | **Palm** | **-** | **-** |  |  | -409.80 | 0.997 | 0.219 | 0.103 | -427 | 0.991 | -10.92 | 0.131 |
|  | **Shrub** | -655.5 | 0.977 |  |  | -410.70 | 0.997 | 0.048 | 0.758 |  |  | -11.38 | 0.127 |
|  | **Tree** | 1988.6 | 0.992 |  |  | -410.30 | 0.997 | -0.007 | 0.924 | 781 | 0.991 | -6.772 | 0.268 |
| Dispersal Mode | **Birds** | 1164.7 | 0.977 | **-** | **-** | -0.369 | 0.307 |  |  |  |  |  |  |
|  | **Hidrochory** | **-** | **-** | **-** | **-** | **-** | **-** | 833.00 | 0.991 |  |  | -2079 | 0.956 |
|  | **Mammals** | 1076.9 | 0.982 | **-** | **-** | -0.638 | 0.151 | 0.023 | 0.775 | 0.65 | 0.230 | -1.808 | 0.540 |
|  | **Mixed** | **-** | **-** | **-** | **-** | 0.224 | 0.577 |  |  |  |  |  |  |
|  | **Wind** | **-** | **-** | 263.0 | 0.994 | -0.588 | 0.194 | 0.079 | 0.366 | -1.01 | 0.107 | -4.312 | 0.171 |
| Pollination Mode | **Beetles** | **-** | **-** | **-** | **-** | -0.093 | 0.790 | 0.053 | 0.677 |  |  | **12.81** | **0.020** |
|  | **Flies** | **-** | **-** | **-** | **-** | **-** | **-** |  |  |  |  |  |  |
|  | **Himenoptera** | **-** | **-** | -126.6 | 0.990 | 584.20 | 0.993 | 0.004 | 0.954 | -1300 | 0.993 | 5.397 | 0.172 |
|  | **Hummingbirds** | **-** | **-** | **-** | **-** | 174.20 | 0.996 | -0.017 | 0.857 |  |  | 7.527 | 0.111 |
|  | **Lepidoptera** | **-** | **-** | **-** | **-** | 584.00 | 0.993 | -0.083 | 0.343 |  |  | 2.415 | 0.571 |
|  | **Wind** | **-** | **-** | **-** | **-** | **-** | **-** |  |  |  |  |  |  |
| Mating system | **Mixed** | **-** | **-** | **-** | **-** | 0.497 | 0.181 |  |  |  |  |  |  |
|  | **Outcrossing** | **-** | **-** | **-** | **-** | 0.286 | 0.341 | -0.006 | 0.903 | -0.13 | 0.725 | 1.942 | 0.402 |
| Breeding system | **Dioecious** |  |  | **-** | **-** | -1.141 | 0.157 |  |  |  |  |  |  |
|  | **Monoecious** | 817.3 | 0.988 | **-** | **-** | -0.814 | 0.128 | -0.052 | 0.572 | -1208 | 0.981 | 3.417 | 0.270 |
|  | **Hermaphrodite** | -1359.6 | 0.974 | **-** | **-** | -0.757 | 0.214 | 0.012 | 0.898 | -1209 | 0.981 | 5.190 | 0.098 |
| Geographic range | **Wide** |  |  | **-** | **-** | 0.324 | 0.106 | 0.069 | 0.207 | 520 | 0.986 |  |  |
| Habitat | **Mangroves** |  |  | **-** | **-** | **-** | **-** | -1122.00 | 0.983 |  |  | 1985 | 0.959 |
|  | **Mixed** |  |  | **-** | **-** | **-** | **-** | -288.70 | 0.995 |  |  | -84.93 | 0.996 |
|  | **Rain forests** | -136.9 | 0.999 | 193.6 | 0.991 | 0.673 | 0.188 | -288.70 | 0.995 |  |  | -87.08 | 0.996 |
|  | **Rocky fields** |  |  | **-** | **-** | **1.73** | **0.038** | -288.40 | 0.995 |  |  | -94.68 | 0.996 |
|  | **Rocky savannas** |  |  | **-** | **-** | **-** | **-** | -288.80 | 0.995 |  |  | -87.24 | 0.996 |
|  | **Savannas** |  |  | 498.6 | 0.993 | 585.00 | 0.993 | -288.70 | 0.995 |  |  | -88.63 | 0.996 |
|  | **Seasonally dry forests** | 2446.7 | 0.969 | 151.5 | 0.978 | 0.729 | 0.176 | -288.70 | 0.995 | 0.91 | 0.136 | -86.66 | 0.996 |
|  | **Wetlands** | **-** | **-** | **-** | **-** | **-** | **-** |  |  |  |  |  |  |
