# Supplementary material for: Pollination Mode and Mating System Explain Patterns in Genetic Differentiation in Neotropical Plants
Source: PLoS One. 2016 Jul 29;11(7):e0158660. doi: 10.1371/journal.pone.0158660 (PMC4966973; doi:10.1371/journal.pone.0158660)
Supplement: S7 Table — h, haplotype diversity π, nucleotide diversity. Significant values are denoted in bold. (DOCX) [file pone.0158660.s008.docx]

**Pollination mode and mating system explains patterns in genetic diversity and differentiation in Neotropical plants**

Liliana Ballesteros-Mejia*^1^*, Natácia E Lima*^1^*, Matheus S. Lima-Ribeiro*^2^*, Rosane G Collevatti*^1^*

**S7 Table.** **Mean values of the posterior distribution of the GLMM for nuclear genome, for genetic parameters***. h*, haplotype diversity π, nucleotide diversity. Significant values are denoted in bold.

| **Class**  **Parameter** |  | ***h*** | | **π** | |  |
| --- | --- | --- | --- | --- | --- | --- |
|  |  | **Mean** | **P-value** | | **Mean** | **P-value** |
| **Growth form** | **Herb** |  |  | |  |  |
|  | **Palm** |  |  | |  |  |
|  | **Shrub** | -1243.70 | 0.975 | | 933.50 | 0.973 |
|  | **Tree** | -195.40 | 0.998 | | 447.20 | 0.996 |
| **Dispersal Mode** | **Birds** |  |  | |  |  |
|  | **Hidrochory** | 196.30 | 0.998 | | -337.50 | 0.984 |
|  | **Mammals** | -1248.50 | 0.996 | | 1376.00 | 0.991 |
|  | **Mixed** | -1243.70 | 0.975 | | 933.50 | 0.973 |
|  | **Wind** | -1707.30 | 0.976 | | 486.30 | 0.990 |
| **Pollination Mode** | **Beetles** |  |  | |  |  |
|  | **Flies** |  |  | |  |  |
|  | **Himenoptera** |  |  | | 442.30 | 0.992 |
|  | **Hummingbirds** |  |  | |  |  |
|  | **Lepidoptera** | 987.70 | 0.976 | | -933.50 | 0.973 |
|  | **Wind** |  |  | |  |  |
| **Mating System** | **Mixed** | 889.00 | 0.989 | | 71.67 | 0.990 |
|  | **Outcrossing** | 1235.00 | 0.997 | | 0.008 | 0.944 |
| **Breeding system** | **Dioecious** |  |  | |  |  |
|  | **Monoecious** | 546.40 | 0.998 | | -1447.00 | 0.989 |
|  | **Hermaphrodite** |  |  | | -486.30 | 0.990 |
| **Geographic range** | **Wide** | -906.40 | 0.987 | | 0.004 | 0.976 |
|  | **Grasslands** |  |  | |  |  |
| **Habitat** | **Mangroves** |  |  | |  |  |
|  | **Mixed** |  |  | |  |  |
|  | **Rain forests** | 898.50 | 0.992 | | -752.20 | 0.997 |
|  | **Rocky fields** |  |  | |  |  |
|  | **Rocky savannas** | 1057.70 | 0.995 | | 65.62 | 0.998 |
|  | **Savannas** |  |  | | -752.20 | 0.997 |
|  | **Seasonally dry forests** | 898.50 | 0.992 | | -752.20 | 0.997 |
|  | **Wetlands** |  |  | |  |  |
