# Supplementary material for: Pollination Mode and Mating System Explain Patterns in Genetic Differentiation in Neotropical Plants
Source: PLoS One. 2016 Jul 29;11(7):e0158660. doi: 10.1371/journal.pone.0158660 (PMC4966973; doi:10.1371/journal.pone.0158660)
Supplement: S8 Table — FST, genetic differentiation among populations; h, haplotype diversity; π, nucleotide diversity. Significant values are denoted in bold and grey-shaded. (DOCX) [file pone.0158660.s009.docx]

**Pollination mode and mating system explains patterns in genetic diversity and differentiation in Neotropical plants**

Liliana Ballesteros-Mejia*^1^*, Natácia E Lima*^1^*, Matheus S. Lima-Ribeiro*^2^*, Rosane G Collevatti*^1^*

**S8 Table.** **Mean values of the posterior distribution of the GLMM for chloroplast genome, for genetic parameters.** *F_ST_*, genetic differentiation among populations; *h*, haplotype diversity; π, nucleotide diversity. Significant values are denoted in bold and grey-shaded.

| Class | Parameter | *F_ST_* | | *h* | | π | |
| --- | --- | --- | --- | --- | --- | --- | --- |
|  |  | **Mean** | **P-value** | **Mean** | **P-value** | **Mean** | **P-value** |
| Growth form | **Herb** | -0.441 | 0.171 | 23.51 | 0.992 |  |  |
|  | **Palm** | 259.8 | 0.995 | 1814.0 | 0.986 | -108.80 | 0.996 |
|  | **Shrub** | **0.647** | **0.040** | -535.0 | 0.998 | -655.10 | 0.979 |
|  | **Tree** | -0.109 | 0.693 | 377.4 | 0.991 | -655.10 | 0.979 |
| Dispersal mode | **Birds** |  |  |  |  |  |  |
|  | **Hidrochory** |  |  | 19.45 | 0.992 | 2148.00 | 0.982 |
|  | **Mammals** | 0.474 | 0.156 | -559.80 | 0.991 | 0.008 | 0.853 |
|  | **Mixed** |  |  |  |  |  |  |
|  | **Wind** | 0.524 | 0.090 | -912.80 | 0.981 | 0.009 | 0.856 |
| Pollination Mode | **Beetles** |  |  |  |  |  |  |
|  | **Flies** |  |  |  |  |  |  |
|  | **Himenoptera** | 0.216 | 0.249 | 0.02 | 0.915 | -0.011 | 0.712 |
|  | **Hummingbirds** | 0.024 | 0.957 |  |  | -0.016 | 0.671 |
|  | **Lepidoptera** | 0.190 | 0.510 | 0.0024 | 0.985 | -0.013 | 0.764 |
|  | **Wind** |  |  |  |  |  |  |
| Mating System | **Mixed** | -0.214 | 0.291 | 210.30 | 0.995 | -0.001 | 0.982 |
|  | **Outcrossing** | -0.155 | 0.375 | -429.90 | 0.997 | 0.008 | 0.750 |
| Breeding system | **Dioecious** |  |  |  |  |  |  |
|  | **Monoecious** | -0.133 | 0.742 | -38.30 | 0.991 | 0.002 | 0.967 |
|  | **Hermaphrodite** | -0.183 | 0.658 | 247.10 | 0.997 | -0.005 | 0.910 |
| Geographic range | **Wide** | 0.279 | 0.088 | 262.20 | 0.985 | 0.003 | 0.911 |
| Habitat | **Grasslands** | 0.106 | 0.788 | 431 | 0.997 |  |  |
|  | **Mangroves** |  |  | -1758 | 0.982 | -1493.00 | 0.987 |
|  | **Mixed** |  |  | -186 | 0.996 | 655.20 | 0.979 |
|  | **Rain forests** | -0.645 | 0.120 | 430 | 0.997 | -0.001 | 0.950 |
|  | **Rocky fields** | -0.002 | 0.997 | 349 | 0.989 | 655.20 | 0.979 |
|  | **Rocky savannas** | -0.627 | 0.191 | 374 | 0.996 | 655.10 | 0.979 |
|  | **Savannas** | -0.350 | 0.473 | -186 | 0.996 | 655.10 | 0.979 |
|  | **Seasonally dry forests** | -0.686 | 0.114 | -1728 | 0.988 | 108.80 | 0.996 |
|  | **Wetlands** | -260.5 | 0.995 | 431 | 0.997 | -1493.00 | 0.987 |
