# Supplementary material for: Pollination Mode and Mating System Explain Patterns in Genetic Differentiation in Neotropical Plants
Source: PLoS One. 2016 Jul 29;11(7):e0158660. doi: 10.1371/journal.pone.0158660 (PMC4966973; doi:10.1371/journal.pone.0158660)
Supplement: S9 Table — Significant values are denoted in bold. FST, genetic differentiation among populations; HeS, mean genetic diversity among populations, He, genetic diversity within population; FIS, inbreeding coefficient. SE, standard error. (DOCX) [file pone.0158660.s010.docx]

**Pollination mode and mating system explains patterns in genetic diversity and differentiation in Neotropical plants**

Liliana Ballesteros-Mejia*^1^*, Natácia E Lima*^1^*, Matheus S. Lima-Ribeiro*^2^*, Rosane G Collevatti*^1^*

**S9 Table.** **Phylogenetic generalized least squares for growth form, for each genetic parameter analyzed.** Significant values are denoted in bold. *F_ST_*, genetic differentiation among populations; *He_S_*, mean genetic diversity among populations, *He*, genetic diversity within population; *F_IS_*, inbreeding coefficient. SE, standard error.

| **Parameter** | ***F_ST_* (microsatellite markers)** | | | ***F_IS_* (isozymes markers)** | | | ***He_S_* (Dominant markers)** | | | ***He_S_* (microsatellite markers)** | | | ***AR*(microsatellite markers)** | | |
| --- | --- | --- | --- | --- | --- | --- | --- | --- | --- | --- | --- | --- | --- | --- | --- |
| **Variable** | **Coefficient ±SE** | **t-value** | **P-value** | **Coefficient ±SE** | **t-value** | **P-value** | **Coefficient ±SE** | **t-value** | **P-value** | **Coefficient ±SE** | **t-value** | **P-value** | **Coefficient ±SE** | **t-value** | **P-value** |
| **Intercept** | 0.22±0.24 | 0.91 | 0.37 | 0.17±0.32 | 0.52 | 0.60 | 0.03±0.21 | 0.16 | 0.88 | 0.52±0.23 | 2.25 | 0.03 | 7.25±4.36 | 1.66 | 0.11 |
| **Herb** | -0.26±0.31 | -0.86 | 0.39 | 0.16±0.37 | 0.42 | 0.68 | 0.37±0.21 | 1.80 | 0.10 | 0.21±0.31 | 0.69 | 0.50 | 0.97±5.52 | 0.18 | 0.86 |
| **Palm** | -0.06±0.22 | -0.25 | 0.81 | -0.51±0.26 | -1.99 | 0.06 | 0.16±0.26 | 0.63 | 0.54 | 0.28±0.21 | 1.33 | 0.19 | -2.62±3.72 | -0.70 | 0.49 |
| **Shrub** |  |  |  | -0.10±0.30 | -0.34 | 0.74 | 0.14±0.20 | 0.69 | 0.50 | -0.04±0.30 | -0.14 | 0.89 | 1.38±5.66 | 0.24 | 0.81 |
| **Tree** | -0.03±0.26 | -0.10 | 0.92 | -0.07±0.33 | -0.21 | 0.84 | 0.15±0.26 | 0.57 | 0.58 | 0.20±0.24 | 0.81 | 0.42 | 5.07±4.59 | 1.11 | 0.28 |

| **Parameter** | ***F_ST_* (Chloroplast markers)** | | |
| --- | --- | --- | --- |
| **Variable** | **Coefficient ±SE** | **t-value** | **P-value** |
| **Intercept** | 0.46±0.31 | 1.45 | 0.15 |
| **Herb** | 0.39±0.35 | 1.12 | 0.27 |
| **Palm** | 0.34±0.33 | 1.03 | 0.31 |
| **Shrub** | 0.54±0.37 | 1.46 | 0.15 |
| **Tree** | 0.21±0.31 | 0.67 | 0.51 |
