# Supplementary material for: Pollination Mode and Mating System Explain Patterns in Genetic Differentiation in Neotropical Plants
Source: PLoS One. 2016 Jul 29;11(7):e0158660. doi: 10.1371/journal.pone.0158660 (PMC4966973; doi:10.1371/journal.pone.0158660)
Supplement: S10 Table — Significant values are denoted in bold. FST, genetic differentiation among populations; HeS, mean genetic diversity among populations, He, genetic diversity within population; FIS, inbreeding coefficient. SE, standard error. (DOCX) [file pone.0158660.s011.docx]

**Pollination mode and mating system explains patterns in genetic diversity and differentiation in Neotropical plants**

Liliana Ballesteros-Mejia*^1^*, Natácia E Lima*^1^*, Matheus S. Lima-Ribeiro*^2^*, Rosane G Collevatti*^1^*

**S10 Table.** **Phylogenetic generalized least squares for dispersal mode, for each genetic parameter analyzed.** Significant values are denoted in bold. *F_ST_*, genetic differentiation among populations; *He_S_*, mean genetic diversity among populations, *He*, genetic diversity within population; *F_IS_*, inbreeding coefficient. SE, standard error.

| **Parameter** | ***F_ST_* (microsatellite markers)** | | | ***F_IS_* (isozymes markers)** | | | ***He_S_* (Dominant markers)** | | | ***He_S_* (microsatellite markers)** | | | ***AR*(microsatellite markers)** | | |
| --- | --- | --- | --- | --- | --- | --- | --- | --- | --- | --- | --- | --- | --- | --- | --- |
| **Variable** | **Coefficient ±SE** | **t-value** | **P-value** | **Coefficient ±SE** | **t-value** | **P-value** | **Coefficient±SE** | **t-value** | **P-value** | **Coefficient ±SE** | **t-value** | **P-value** | **Coefficient ±SE** | **t-value** | **P-value** |
| **Intercept** | 0.23±0.16 | 1.48 | 0.15 | 0.27±0.31 | 0.89 | 0.38 | 0.12±0.16 | 0.76 | 0.46 | 0.71±0.15 | 4.80 | 0.00 | 10.61±2.78 | 3.82 | 0.00 |
| **Birds** |  |  |  | -0.18±0.28 | -0.65 | 0.52 |  |  |  |  |  |  |  |  |  |
| **Hidrochory** | 0.30±0.17 | 1.80 | 0.08 |  |  |  | 0.07±0.21 | 0.33 | 0.75 | -0.21±0.24 | -0.90 | 0.38 | -3.92±4.28 | -0.91 | 0.37 |
| **Mammals** | -0.07±0.12 | -0.58 | 0.57 | -0.19±0.25 | -0.76 | 0.45 | 0.23±0.13 | 1.83 | 0.09 | -0.02±0.10 | -0.24 | 0.81 | 0.85±2.05 | 0.41 | 0.68 |
| **Mixed** |  |  |  | -0.16±0.36 | -0.46 | 0.65 |  |  |  |  |  |  |  |  |  |
| **Wind** | -0.10±0.11 | -0.94 | 0.36 | -0.15±0.27 | -0.54 | 0.60 | 0.23±0.18 | 1.27 | 0.23 | 0.03±0.10 | 0.26 | 0.80 | -1.87±2.03 | -0.92 | 0.37 |

| **Parameter** | ***F_ST_* (Chloroplast markers)** | | |
| --- | --- | --- | --- |
| **Variable** | **Coefficient ±SE** | **t-value** | **P-value** |
| **Intercept** | 0.63±0.27 | 2.33 | 0.02 |
| **Birds** |  |  |  |
| **Hidrochory** |  |  |  |
| **Mammals** | 0.21±0.21 | 0.99 | 0.33 |
| **Mixed** |  |  |  |
| **Wind** | 0.07±0.18 | 0.40 | 0.69 |
