# Supplementary material for: Pollination Mode and Mating System Explain Patterns in Genetic Differentiation in Neotropical Plants
Source: PLoS One. 2016 Jul 29;11(7):e0158660. doi: 10.1371/journal.pone.0158660 (PMC4966973; doi:10.1371/journal.pone.0158660)
Supplement: S11 Table — Significant values are denoted in bold. FST, genetic differentiation among populations; HeS, mean genetic diversity among populations, He, genetic diversity within population; FIS, inbreeding coefficient. SE, standard error. (DOCX) [file pone.0158660.s012.docx]

**Pollination mode and mating system explains patterns in genetic diversity and differentiation in Neotropical plants**

Liliana Ballesteros-Mejia*^1^*, Natácia E Lima*^1^*, Matheus S. Lima-Ribeiro*^2^*, Rosane G Collevatti*^1^*

**S11 Table.** **Phylogenetic generalized least squares for breeding system, for each genetic parameter analyzed.** Significant values are denoted in bold. *F_ST_*, genetic differentiation among populations; *He_S_*, mean genetic diversity among populations, *He*, genetic diversity within population; *F_IS_*, inbreeding coefficient. SE, standard error.

| **Parameter** | ***F_ST_* (microsatellite markers)** | | | ***F_IS_* (isozymes markers)** | | | ***He_S_* (Dominant markers)** | | | ***He_S_* (microsatellite markers)** | | | ***AR*(microsatellite markers)** | | |
| --- | --- | --- | --- | --- | --- | --- | --- | --- | --- | --- | --- | --- | --- | --- | --- |
| **Variable** | **Coefficient ±SE** | **t-value** | **P-value** | **Coefficient ±SE** | **t-value** | **P-value** | **Coefficient±SE** | **t-value** | **P-value** | **Coefficient ±SE** | **t-value** | **P-value** | **Coefficient ±SE** | **t-value** | **P-value** |
| **Intercept** | 0.09±0.21 | 0.44 | 0.66 | 0.32±0.28 | 1.17 | 0.25 | 0.31±0.13 | 2.48 | 0.03 | 0.74±0.20 | 3.77 | 0.00 | 7.87±3.28 | 2.40 | 0.02 |
| **Dioecious** | - | - | - | -0.28±0.25 | -1.13 | 0.27 | - | - | - | - | - | - | - |  |  |
| **Monoecious** | 0.13±0.14 | 0.89 | 0.38 | -0.31±0.28 | -1.11 | 0.28 | -0.09±0.22 | -0.41 | 0.69 | 0.13±0.13 | 0.94 | 0.35 | 1.10±3.21 | 0.34 | 0.73 |
| **Hermaphrodite** | 0.09±0.17 | 0.49 | 0.63 | -0.21±0.21 | -0.98 | 0.34 | - | - | - | -0.05±0.16 | -0.32 | 0.75 | 3.27±2.57 | 1.27 | 0.21 |

| **Parameter** | ***F_ST_* (Chloroplast markers)** | | |
| --- | --- | --- | --- |
| **Variable** | **Coefficient ±SE** | **t-value** | **P-value** |
| **Intercept** | 0.58±0.29 | 2.00 | 0.05 |
| **Dioecious** | - | - | - |
| **Monoecious** | 0.29±0.29 | 0.97 | 0.34 |
| **Hermaphrodite** | 0.16±0.22 | 0.72 | 0.48 |
